# Structural and functional analysis of EntV reveals a 12 amino acid fragment protective against fungal infections

**DOI:** 10.1038/s41467-022-33613-1

**Published:** 2022-10-13

**Authors:** Melissa R. Cruz, Shane Cristy, Shantanu Guha, Giuseppe Buda De Cesare, Elena Evdokimova, Hiram Sanchez, Dominika Borek, Pedro Miramón, Junko Yano, Paul L. Fidel, Alexei Savchenko, David R. Andes, Peter J. Stogios, Michael C. Lorenz, Danielle A. Garsin

**Affiliations:** 1 Department of Microbiology and Molecular Genetics, The University of Texas Health Science Center at Houston, Houston, TX 77030 USA; 2 BioZone, Department of Chemical Engineering and Applied Chemistry, University of Toronto, Toronto, ON M5S 3E5 Canada; 3 Department of Medicine, University of Wisconsin, Madison, WI 53705 USA; 4 Department of Medical Microbiology and Immunology, University of Wisconsin, Madison, WI 53705 USA; 5 Department of Biophysics, The University of Texas Southwestern Medical Center, Dallas, TX 75390 USA; 6 Department of Biochemistry, The University of Texas Southwestern Medical Center, Dallas, TX 75390 USA; 7 Department of Oral and Craniofacial Biology, Louisiana State University Health School of Dentistry, New Orleans, LA 70119 USA; 8 Department of Microbiology, Immunology and Infectious Diseases, University of Calgary, Calgary, AB T2N 4N1 Canada; 9 Center for Structural Genomics of Infectious Diseases (CSGID), Chicago, IL USA

**Keywords:** Fungal pathogenesis, Bacterial structural biology, Fungal infection

## Abstract

Fungal pathogens are a continuing challenge due to few effective antifungals and a rise in resistance. In previous work, we described the inhibition of *Candida albicans* virulence following exposure to the 68 amino acid bacteriocin, EntV, secreted by *Enterococcus faecalis*. Here, to optimize EntV as a potential therapeutic and better understand its antifungal features, an X-ray structure is obtained. The structure consists of six alpha helices enclosing a seventh 16 amino acid helix (α7). The individual helices are tested for antifungal activity using in vitro and nematode infection assays. Interestingly, α7 retains antifungal, but not antibacterial activity and is also effective against *Candida auris* and *Cryptococcus neoformans*. Further reduction of α7 to 12 amino acids retains full antifungal activity, and excellent efficacy is observed in rodent models of *C. albicans* oropharyngeal, systemic, and venous catheter infections. Together, these results showcase EntV-derived peptides as promising candidates for antifungal therapeutic development.

## Abstract (web summary)

*Enterococcus faecalis* has been reported to inhibit *Candida albicans* virulence via secretion of the bacteriocin EntV. Here, the authors present the crystal structure and characterise the antifungal properties of this peptide in numerous in vitro and in vivo assays.

## Introduction

Management of disseminated fungal infections continues to be a major clinical problem. The limited spectrum of available antifungal agents contributes to both the development of acquired resistance and the rise in incidence of previously rare but intrinsically resistant pathogens^1,2^. Consequently, there is an unacceptably high mortality rate in patients with fungal infections^3^. Antifungal discovery is complicated by the similar cell biology of fungi and mammals; as a result, there are only three classes of drugs for systemic infections, the newest of which was discovered nearly 40 years ago and approved for clinical use in 2001^4^. This illustrates the challenges of developing new antifungals, despite the dire need for them.

Life-threatening fungal infections are seen nearly exclusively in immunodeficient patients, with healthy individuals routinely exposed to these pathogens^5–7^. *Candida albicans* is archetypal: it is a ubiquitous mammalian commensal of the gastrointestinal and urogenital tracts as well as the skin and is rarely isolated from environmental sources^8^. The normally benign association of most *C. albicans* with the human host raises the prospect that successful antifungal therapy need not involve eradication, but rather a restoration of microbial balance, so as to confine it to a non-pathogenic association. Consequently, significant effort has been expended to understand the normal and pathological interactions of *C. albicans* with the host^9^. Less attention has been paid to how these interactions may be impacted by other components of the microbiome, but *C. albicans* has been observed to have both synergistic and antagonistic relationships with various bacterial species, including human pathogens such as *Staphylococcus aureus*, *Pseudomonas aeruginosa, Streptococcus mutans* and *Enterococcus faecalis*^10–12^. It stands to reason that *C. albicans*, often considered to be an obligate mammalian commensal, would have evolved specific responses to its bacterial neighbors in the microbiome and is thus a platform for uncovering molecular signals that may have therapeutic potential.

We previously reported that the Gram-positive bacterium *E. faecalis* antagonizes the virulence of *C. albicans*^13,14^. These two opportunistic species share common host niches and are perhaps the most common bacterial–fungal pairing in polymicrobial infections, suggesting that they may cooperate to promote either commensal or pathogenic behaviors^10,15^. The effect on *C. albicans* is in part mediated by EntV, a bacteriocin produced by *E. faecalis* as a pre-pro-peptide^14,16,17^. Processing includes removal of a signal peptide, formation of a disulfide bond, and cleavage by an extracellular protease^17,18^. The processed, active form of EntV is 68 amino acids and is both necessary and sufficient to inhibit *C. albicans* biofilm formation in vitro and pathogenesis in both a nematode (*Caenorhabditis elegans*) infection model and a mouse model of oropharyngeal candidiasis (OPC)^14^. Notably, EntV appears to have no fungicidal or fungistatic action, but works to inhibit adhesion and hyphal morphogenesis, both of which are required for virulence in this species^14^. EntV is thus a candidate anti-virulence agent.

There are several significant obstacles to further development of EntV as an antifungal agent in its native form. Chemical synthesis of a 68 amino acid protein that contains a required disulfide bond is nontrivial and attempts to produce this recombinantly in bacteria have not been successful due to toxicity^14^. This is likely due to its bacteriocin activity, which poses the potential for off-target effects on the bacterial microbiome^16,17^. Thus, we sought to further investigate the structural determinants of the antifungal activity of EntV and whether we could reduce the size and complexity of the active moiety and separate the bacteriocin and antifungal functions.

Here, the three-dimensional structure of the secreted form of EntV prior to extracellular cleavage is reported. By functional analysis one alpha helix with significant antifungal but no bacteriocin activity is identified. Moreover, shorter fragments of the helix, down to 12 amino acids, exhibited full antifungal activity at nanomolar concentrations in in vitro assays and animal models of candidiasis, with a 10mer variant retaining some function. These peptides have promise as broad-spectrum antifungal agents as we showed they are active against at least some strains of *Cryptococcus neoformans* and the multi-drug resistant *Candida auris*. Thus, EntV-derived peptides are candidates for development of anti-virulence agents for the treatment or prevention of fungal infections.

## Results

### “Clasping palms” structure of EntV

**Fig. 1:**
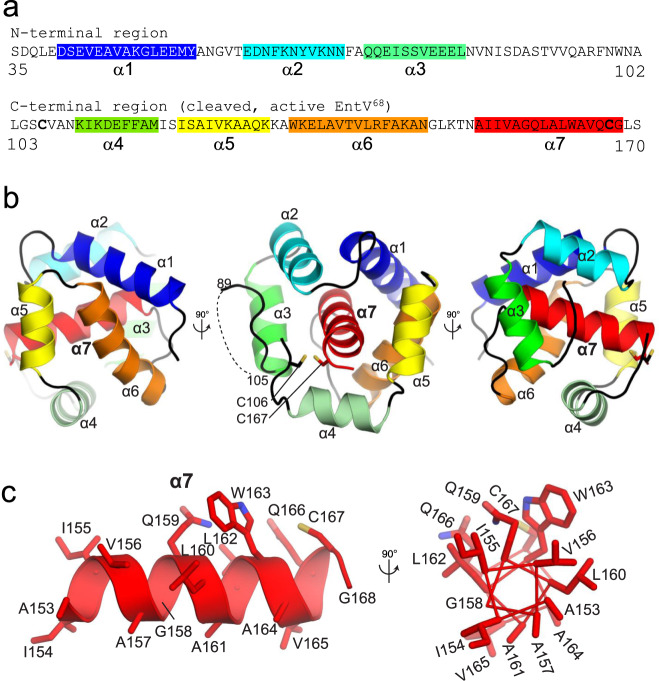
The sequence and structure of EntV. **a** The sequence of full-length, secreted EntV^136^. The second line is EntV^68^, the active form of the protein generated following cleavage. The highlighted sequences and their colors correspond to the alpha helices resolved in the X-ray structure shown in **b**. **c** The structure and composition of the central α7 helix.

EntV was previously characterized as a secreted protein that undergoes multiple processing events. The 170 amino acid translated protein is directed to the secretion system by a signal sequence at its N-terminus. The signal peptide is cleaved during secretion releasing a 136 amino acid protein (Fig. 1a)^16–18^. The protein is further cleaved in half by the extracellular protease GelE, generating the active form of EntV which comprises the 68 amino acid C-terminus^14,17,18^. A disulfide bond formed between two cysteines in the C-terminus (C106 and C167 in Fig. 1a) was also shown to be necessary for activity^18^.

To better understand the basis for the antimicrobial properties of EntV, we sought to visualize its structure by X-ray crystallography. Unprocessed EntV^136^ was recombinantly purified from an *E. coli* strain as a selenomethionine-derivatized protein and crystallized. The structure was determined using single anomalous dispersion (SAD) phasing to 1.8 Å resolution, allowing for high resolution analysis of the structure. All residues were visible in the electron density except for the first two amino acids^19,20^, the last two (169–170) and an internal region corresponding to residues 90–104; this latter region notably corresponds to the GelE cleavage site, and we postulate that there is natural flexibility in this region. The predicted disulfide bond between C106 and C167 was not observed in the structure due to the necessity of producing the protein under reducing conditions in the *E. coli* cytosol. However, the two cysteine residues are co-localized within 3.4 Å, consistent with the ability to form an intramolecular disulfide bond. Interestingly, the structure resembled two “clasping palms” comprised of three α-helices each (Fig. 1b). The first three helices are in the N-terminus of EntV whereas the second three are part of the C-terminus. The two palms enclose a final C-terminal helix, α7 (Fig. 1a, b). We noticed a strong charge asymmetry between the external faces of the two palms (Supplementary Fig. 1a). The NTD (N-terminal domain) external face is negatively charged, while the CTD (C-terminal domain) external face is positively charged. The positive charge of the external face of the CTD is due to the presence of many lysine and arginine residues, while in contrast, the interior face that interacts with α7 is hydrophobic/neutral (Supplementary Fig. 1b). α7 itself is strongly hydrophobic, as 11 of its 16 residues are hydrophobic sidechains (Fig. 1c). These observations are consistent with the sheltering of the hydrophobic α7 in the interior of the EntV^136^ structure.

### The α7 helix is sufficient for antifungal activity

**Fig. 2:**
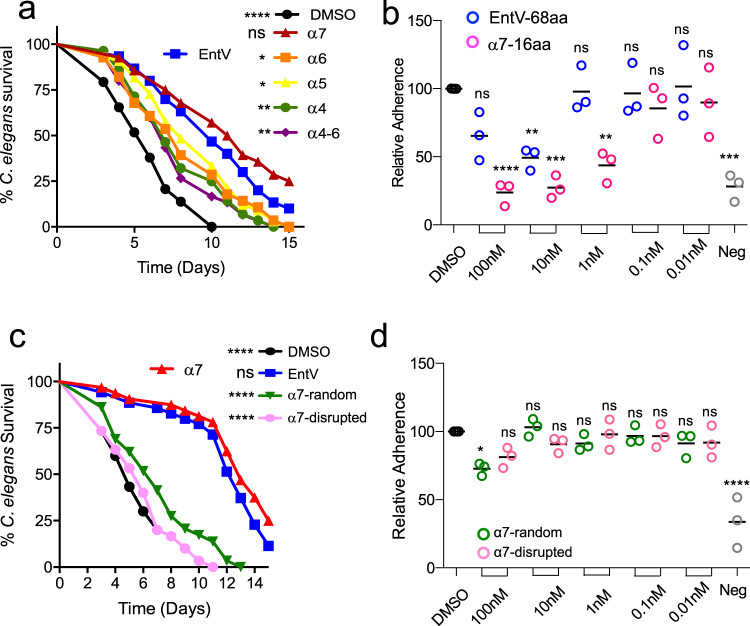
The α7 helix of EntV has antifungal activity. **a**, **c** show survival over time of *C. elegans* infected with *C. albicans* and exposed to 1 nM of the indicated fragments of EntV based on the structure in Fig. 1a or versions of the α7 helix, respectively. Random sequence = TNVGALWICGLAVIQAAQLS and disrupted sequence = TNAAAAQQGGIICLLVVWLS. Statistical differences in survival were compared to animals in the exposure condition on the left of each legend by Mantel–Cox log rank analysis. An n of 60 animals was used and one representative trial is shown. Exact *p* values from top to bottom: **a** <0.0001, 0.1900, 0.0361, 0.0583, 0.0059, 0.0019. **c** <0.0001, 0.1666, <0.0001, <0.0001. Median survival and *p* values of all trials are shown in Supplementary Data 1. **b**, **d** show adhesion of *C. albicans* to tissue-culture treated plates following incubation with different concentrations of the indicated peptides. “Neg” is a control non-adherent strain (∆*efg1* ∆*cph1*). Lines with error bars indicate the mean and the SD following normalization against the mean of the vehicle control group. Statistical significance in comparison to the DMSO control group was determined using one-way ANOVA followed by Dunnett’s multiple comparison test. An n of three biological replicates, each with six technical replicates, was used. Exact *p* values from left to right are as follows: **b** 0.1065, <0.0001, 0.0071, 0.0001, 0.9998, 0.0026, 0.9997, 0.8961, 0.9998, 0.9875, 0.0002, **d** 0.0101, 0.1223, 0.9994, 0.8044, 0.8456, 0.9996, 0.9994, 0.993, 0.8957, <0.0001.

Based on the solved structure, we tested fragments of EntV for structural moieties that retained antifungal activity. We generated synthetic peptides of each of the four alpha helices (plus two flanking amino acids) from the active C-terminus of EntV––α4, α5, α6 and α7 (Fig. 1a) as well as an α4-α6 construct. The peptides were tested for their ability to protect *C. elegans* against *C. albicans* infection as established in previous work^13,14^. As shown in Fig. 2a, the α7 fragment at a concentration of 1 nM protected *C. elegans* from *C. albicans* infection as well or better than full-length, active EntV^68^ (Fig. 2a and Supplementary Data 1). The other peptide fragments also showed modest protection.

EntV was originally characterized for its antibacterial activity against some Gram-positive species^16,17^. Therefore, the peptides were additionally tested for their ability to inhibit the growth of *Lactobacillus sakei* in liquid culture. As shown in Supplementary Fig. 2, EntV abrogated the growth of *L. sakei* at concentrations as low as 10 nM. However, none of the EntV fragments had any activity in this assay at any of the concentrations tested. These data suggest that the antibacterial and antifungal effects of EntV have different mechanisms and may be separable.

We previously demonstrated that the full-length EntV reduces fungal burden and invasion in a mouse model of oropharyngeal candidiasis (OPC)^14^. This, like many manifestations of *Candida* infections, requires adhesion of the fungal cell to surfaces, both biotic and abiotic, whereupon biofilm structures are commonly formed. We used a model in which binding to plastic surfaces is a measure of the initial stage of biofilm differentiation. In this assay, cells are incubated in polystyrene 96 well plates with the peptides in PBS for one hour and then overlaid with artificial saliva media for 90 min before washing and quantitation of adhesion using crystal violet. Full-length EntV significantly reduced adhesion to polystyrene (Fig. 2b). The core 16aa α7 helix by itself had further increased efficacy, with significant activity remaining at 1 nM (Fig. 2b). The magnitude of the reduction was equivalent that of the negative control (‘neg’ in Fig. 2b), a well-characterized non-adherent and non-hyphal mutant lacking the *CPH1* and *EFG1* transcriptional regulators^21^.

We next designed variants of the α7 peptide to probe the elements that were required for activity. To test if the specific sequence and/or the helical structure were important for enabling activity, we randomized the sequence of α7 in two ways. First, using the peptide folding prediction program PEP-FOLD 3^22–24^, we generated peptides in which the sequence was randomized in a manner predicted to either retain or disrupt the helical structure. Neither peptide provided any significant protection in the worm assay (Fig. 2c), suggesting sequence specificity to the observed activity. Consistent with this, the scrambled peptides also largely lost the ability to inhibit adhesion (Fig. 2d). Thus, the antifungal activity results from the specific sequence and not a generic helical motif.

We observed that while most of the residues that comprise the 16 amino acid core of α7 are hydrophobic, the three polar residues (two glutamines and one cysteine) are on the same face of the helix as shown in a helical wheel projection (Fig. 1c). This cysteine forms a disulfide bond that is required for the activity of the 68 amino acid peptide^16,18^. We speculated that the polar nature of the glutamines might be important and synthesized peptides in which these were both changed either to non-polar isoleucine or to charged glutamic acid. Surprisingly, the isoleucine substitution had no effect on activity in the worm or adhesion assays. In contrast, the glutamic acid variant was notably less effective (Supplementary Fig. 3a, b). Thus, the antifungal activity does not require the helix to be amphipathic.

### Shorter peptides retain activity

**Fig. 3:**
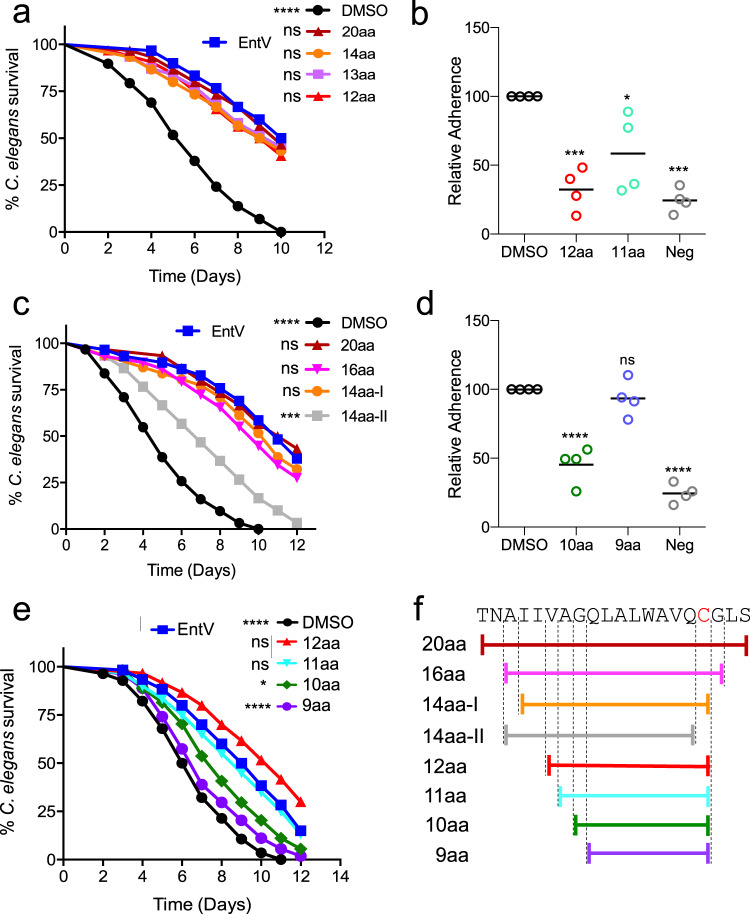
Shorter variants of α7 retain antifungal activity. **a**, **c**, **e** show survival over time of *C. elegans* infected with *C. albicans* and exposed to 1 nM of the indicated α7 fragment as shown in **f**. Statistical differences in survival were compared to animals in the exposure condition on the left of each legend by Mantel–Cox log rank analysis. An n of 60 was used and one representative trial is shown. Exact *p* values from top to bottom: **a** <0.0001, 0.7721, 0.4591, 0.5702, 0.3797, **c** <0.0001, 0.7958, 0.2951, 0.5476, 0.0002. **e** <0.0001, 0.0489, 0.5844, 0.0127, <0.0001. Median survival and *p* values of all trials are shown in Supplementary Data 1. **b**, **d** show adhesion of *C. albicans* to tissue-culture treated plates following incubation with 10 nM of the α7 fragment size variants. “Neg” is the background observed with a non-adherent strain (*∆efg1∆cph1*). Lines with error bars indicate the mean and the SD following normalization against the mean of the vehicle control group. Statistical significance in comparison to the DMSO control group was determined using one-way ANOVA followed by Dunnett’s multiple comparison test. An n of four biological replicates, each with six technical replicates, was used. Exact p values from left to right: **b** 0.0003, 0.0210, 0.0001, **d** <0.0001, 0.6890, <0.0001.

To further understand the features of α7 necessary for its activity, we sought to identify the minimal length that still retained antifungal properties. We iteratively generated peptides of decreasing length and found that peptides down to 12 amino acids retained full activity in the nematode and adhesion assays (Fig. 3a, b, Supplementary Data 1, Supplementary Fig. 3). Two 14 amino acid peptides were tested, one of which contained the cysteine residue (14aa-I; Fig. 3c) and one that did not (14aa-II). Removing the cysteine greatly reduced protection in the worm model. Additionally, mutating the cysteine to a serine in the 12aa peptide abrogated activity (Supplementary Fig. 3c and Supplementary Data 1). However, alkylating the cysteine had no effect, suggesting that the reactivity of the cysteine sulfhydryl is not required. Further reducing the peptide size to 11 or 10 amino acids attenuated activity somewhat in both assays, while a 9-mer peptide was inactive (Fig. 3b, d, e, Supplementary Data 1, Supplementary Fig. 4). The tested peptides are illustrated in Fig. 3f. These data suggest that a 12mer of the α7 helix, retaining a full three turns of the α-helix and ending with cysteine-167, is as effective in protecting against *C. albicans* infection in the worm model and inhibiting surface adhesion as full-length EntV.

### EntV peptides have activity against other fungal species and strains

**Fig. 4:**
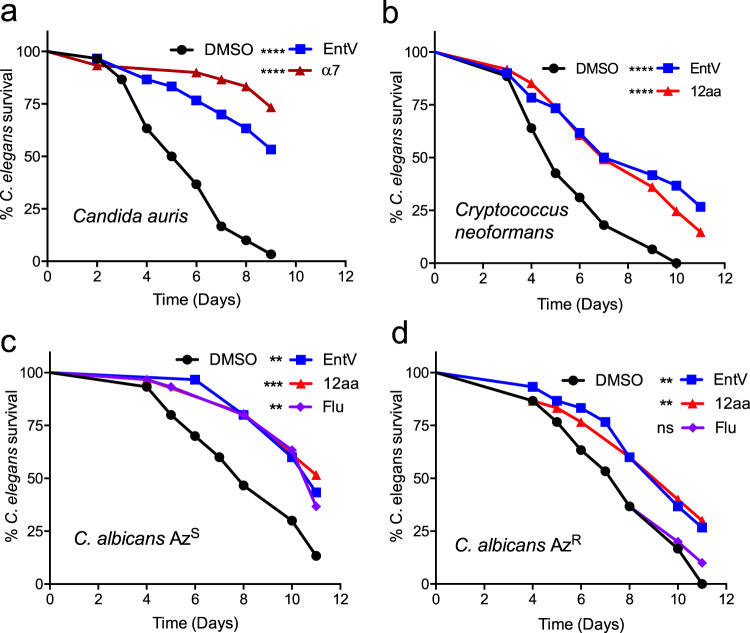
EntV peptides have activity against other fungal species and strains. Survival over time of *C. elegans* infected with *C. auris*, **a***, C. neoformans*, **b**, and *C. albicans* paired clinical isolates before (Az^S^) and after gaining fluconazole resistance (Az^R^), **c**. Peptides were added at 1 nM and fluconazole at 4 ug/ml. Statistical differences in survival were compared to animals to the DMSO vehicle control by Mantel–Cox log rank analysis. An n of 60 was used and one representative trial is shown. Exact *p* values from top to bottom: (**a**) <0.0001, <0.0001, **b** <0.0001, <0.0001, **c** 0.0013, 0.0004, 0.0035, **d** 0.0050, 0.0040, 0.5539. Median survival and *p* values of all trials are shown in Supplementary Data 1.

We previously reported that EntV^68^ inhibited biofilm formation of other *Candida* species including *C. tropicalis, C. parapsiolosis* and *C. glabrata*, suggesting its activity may not be limited to *C. albicans*^14^. Based on these observations, whether EntV and derivative peptides protected *C. elegans* from other fungal species and drug resistant strains of *C. albicans* was tested*. Candida auris* has been defined by the CDC as an emerging pathogen of concern, causing infection in immunocompromised patients that are hard to treat due to high-levels of intrinsic drug-resistance^25^. As shown in Fig. 4a, treatment of a *C. auris* strain with α7 protected animals from killing. The 12aa version of the α7 fragment also protected *C. neoformans* infected worms from killing (Fig. 4b), a pathogen that is a leading cause of death in HIV/AIDS patients due to fungal meningitis^26^*. C. neoformans* is in the phylum basidiomycota and is thus evolutionarily very distant from Candida species (phylum ascomycota), suggesting that the unknown target of EntV is widely conserved amongst fungi. We also tested paired clinical isolates of *C. albicans* in which fluconazole-resistance had been gained following in-patient evolution^27^. Animals infected with both the original strain as well as the fluconazole-resistant descendent were protected by EntV (Fig. 4c). These results warrant future testing of many more species and strains of fungal pathogens to understand the extent of EntV efficacy.

### The 12mer is protective in a mouse model of oropharyngeal candidiasis (OPC)

**Fig. 5:**
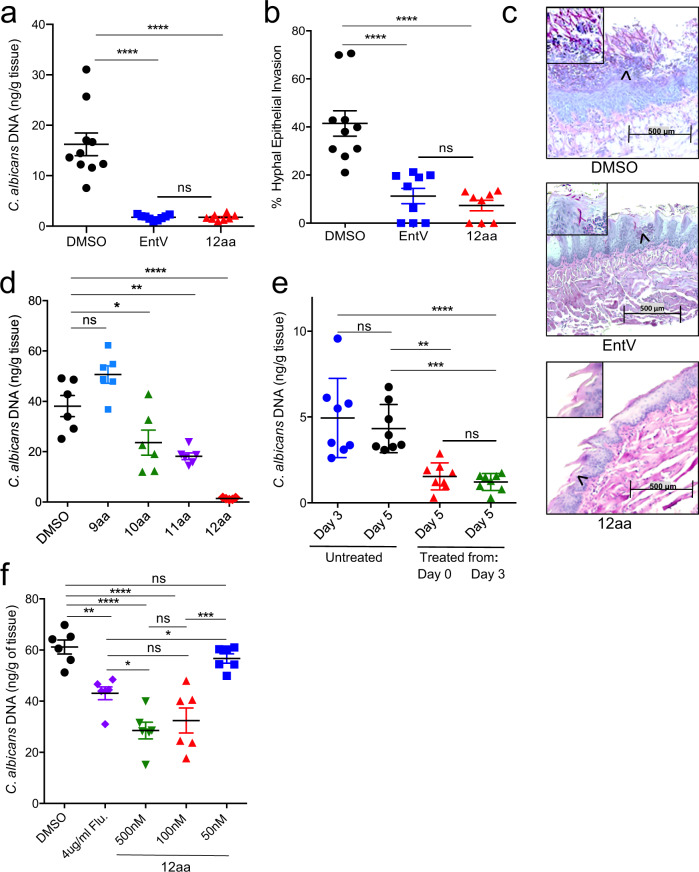
The 12mer is protective in a mouse model of oropharyngeal candidiasis. Amount of *C. albicans* DNA detected by qPCR, **a**, and percentage of the tongue surface showing hyphal invasion, **b**, in untreated animals compared to those given 100 nM EntV and 12aa α7 in their drinking water for 5 days following inoculation. **c** Representative pictures used to score hyphal invasion. Pink staining cells in the areas that are magnified, indicated by the black arrow heads, are *C. albicans*. The cells in the control sample show an elongated, hyphal morphotype more prevalently invading the tissue compared to the treated samples. **d** The amount of *C. albicans* DNA detected by qPCR in animals treated with 100 nM of different fragment lengths of the α7 peptide at day 5. **e** The amount of *C. albicans* DNA detected by qPCR in animals at day 3 or 5 following treatment with 100 nM of the 12mer starting at day 0 or 3. **f** The amount of *C. albicans* DNA detected by qPCR in animals treated with different concentrations of the 12mer in comparison to fluconazole (Flu) at day 5. An n of 6-10 biologically independent animals was used with the exact number indicated by the number of data points. Averages were calculated and the error bars represent the SEM. Horizontal lines mark compared conditions. Significance was determined using one-way ANOVA followed by Tukey’s multiple comparison test. Exact *p* values from top to bottom are as follows: **a** <0.0001, <0.0001, >0.9999, **b** <0.0001, <0.0001, 0.8811, **d** <0.0001, 0.0014, 0.0206, 0.0515, **e** <0.0001, 0.9688, 0.0030, 0.0009, **f** 0.8588, <0.0001, <0.0001, 0.0043, 0.0001, 0.1672, 0.0444, 0.9087, 0.0282.

The 12mer of the α7 helix was tested for efficacy in protecting against *C. albicans* oral infection in mice, a model in which EntV was previously shown to be effective^14^. Briefly, corticosteroid-suppressed mice were orally inoculated with *C. albicans* and provided with water containing vehicle (0.01% DMSO), EntV, or the 12mer peptide (100 nM). After five days, fungal burden was assessed in the tongue using qPCR and epithelial invasion by histological examination. As shown in Fig. 5a, treatment with EntV significantly reduced the fungal burden, as observed in our previous study^14^, and treatment with the 12mer was equally effective. We assessed epithelial invasion of the tongues via histology and determined that the 12 amino acid peptide was at least as effective as the full length EntV (Fig. 5b, c). In contrast, to the extensive hyphal morphology and invasion in control animals, *C. albicans* is found mostly in the yeast morphological state when associated with tissue from the animals treated with EntV or the 12mer (Fig. 5c). Overall, these data show that the 12mer is as effective as EntV^68^ in abrogating an oropharyngeal infection of *C. albicans*.

We also tested the shorter peptides and, consistent with the *C. elegans* and adhesion assays, the peptides of 11 and 10 amino acids reduced fungal burden in the OPC model, but not to the same degree as the 12mer. The 9mer exhibited no significant protection (Fig. 5d). Hyphal invasion was also most reduced following treatment with the 12mer, with some reduction observed in animals exposed to the 10mer and 11mer. The 9mer had no effect (Supplementary Fig. 5a). The results support the conclusion that the 12mer is the optimal fragment length, comparable to or better than EntV^68^ in all assays.

In these experiments, as well as in the previous study, ad libitum treatment with EntV or its truncated variants commenced as soon as the animals recovered from the oral inoculation with *C. albicans* by adding peptide to drinking water. To determine if the 12mer has efficacy as a treatment after the establishment of an infection, we inoculated mice, but did not administer peptide until day three (Fig. 5e). A group of untreated infected animals was also sacrificed at day three to check for successful establishment of the infection. Animals treated with the 12mer starting at day three and sacrificed on day five had significantly less fungal burden compared to both groups of untreated animals. In fact, the level of reduction was not significantly different from those animals who had been treated from day zero (Fig. 5e). The same patterns were observed when hyphal invasion was used as the measurement of infection severity (Supplementary Fig. 5b). These results suggest that the 12mer has efficacy in treating established OPC.

We assessed the efficacy of the 12mer relative to fluconazole, an antifungal agent recommended to treat OPC^28^. At our standard dose of 100 nM, the 12mer was even more effective at reducing fungal burden than 4 µg/mL fluconazole, 8-times the MIC for this strain^29,30^, though this was only statistically significant when we increased the concentration of EntV to 500 nM (Fig. 5f). Hyphal invasion was slightly less prevalent after treatment with 100 nM and 500 nM relative to fluconazole (Supplementary Fig. 5c). We also tested a lower dose (50 nM), which resulted in loss of efficacy in terms of fungal burden, though there was still a significant lowering of hyphal invasion (Fig. 5f and Supplementary Fig. 5c). Overall, these data show that oral treatment with 100 nM 12mer is comparable or better to treatment with fluconazole, a standard and accepted chemotherapeutic.

Given the sustained efficacy of the 12mer to reduce epithelial invasion at 50 nM, we tested yet lower concentrations. At 10 nM and 1 nM there was only a slight reduction in fungal burden on the tongue, but the reduction in invasion remained dramatic (Supplementary Fig. 5d, e). The EntV 12mer appears to promote a commensal, non-invasive, association with the host epithelium at concentrations well below those needed to see a reduction in fungal numbers. Finally, we note that there are no differences in fungal burden, invasion, or EntV/12mer efficacy between male and female mice (Supplementary Fig. 5f, g).

### The 12mer is protective in a rat venous catheter model

**Fig. 6:**
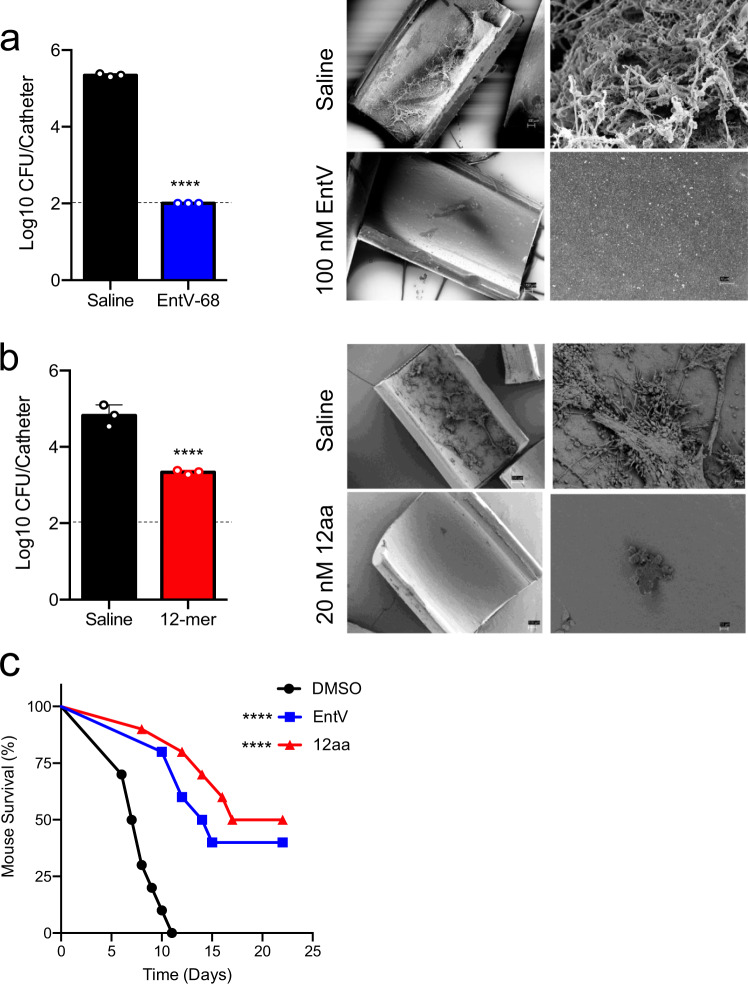
The 12mer is protective in models of venous catheter and systemic *C. albicans* infection. In **a**, **b**, intravenous catheters were implanted into the jugular veins of rats and inoculated with *C. albicans* with or without EntV peptides using an n of three biologically independent animals. After 24 h the catheters were removed and CFUs were determined from one-half (left panels) and scanning electron micrographs were taken at 100x (left) or 1000x (right) of the other half of the catheters (right panels) with **a** showing treatment with EntV at 100 nM and **b** showing treatment with the 12mer at 20 nM. The dashed lines are the detection limit of this assay (100 CFU). The mean of the CFUs were calculated and the error bars represent the standard deviations (SD). An unpaired, two-tailed t test was used to compare the treatments to the controls and the exact *p* values were <0.0001 for both. **c** *C. albicans* was incubated for 2 h in PBS with DMSO, EntV, or the 12mer prior to inoculation via the lateral tail vein. An n of 10 biologically independent animals was used for each group. When moribund, animals were humanely sacrificed. Survival was compared to the DMSO control by Mantel–Cox log rank analysis and the exact *p* values were <0.0001 and <0.0001.

*C. albicans* can form prodigious biofilms on implanted medical devices and the presence of an intravenous catheter is, by itself, a significant risk factor for developing disseminated candidiasis^7,31^. These infections are notoriously difficult to treat and are associated with the drug resistance characteristic of biofilms^32^. We initially tested the full-length EntV in a model in which an intravenous catheter is implanted into the jugular vein of rats and inoculated with *C. albicans* with or without the peptide. After 24 h, the catheter is removed and sectioned to assess fungal burden by plating for CFUs and by scanning electron microscopy (SEM)^33^. Catheters treated with 100 nM EntV were essentially sterile, with fungal burden below the limit of detection (Fig. 6a). As we defined the minimal peptide length, we again utilized this model using the 12mer peptide, this time at a lower concentration to probe the extent of protection. Even as low as 20 nM, the 12mer reduced fungal burdens by 1.5 logs (Fig. 6b) and SEMs showed the catheter surfaces to be largely clear of fungal biofilm (Fig. 6a, b). The data suggest that the EntV peptides are very effective in preventing *C. albicans* biofilm formation on abiotic surfaces and has potential as a treatment and/or a preventative of *C. albicans* medical device-associated infections.

### The 12mer is protective in a systemic mouse model

Systemic, bloodstream infections by *C. albicans* are the most life-threatening to patients. Mortality rates from disseminated candidiasis exceed 40% and new treatments are in dire need. Since EntV impairs hyphal morphogenesis and biofilm formation rather than killing *C. albicans*^14^, it was unclear if EntV and EntV derivatives would be protective in a systemic infection model. Before injection of the 12mer, toxicity and stability of the 12mer was examined. Incubation of EntV and the 12mer caused no lysis of red blood cells (Supplementary Fig. 6a). However, they were not particularly stable in serum; about 10% of the 12mer was detectable following a 24-hour incubation (Supplementary Fig. 6b). To increase the chances of observing an effect, we pre-incubated *C. albicans* with 100 nM of EntV or the 12mer for two hours prior to intravenous (IV) injection of 1 ×10^5^ CFU. CFUs were measured both before and after the two-hour incubation and there was no significant difference, indicating that the EntV versions were not toxic to *C. albicans*. The median survival of animals injected with *C. albicans* alone was 7.5 days and all animals had expired by 11 days. In striking contrast, animals injected with *C. albicans* that had been pre-incubated with EntV or the 12mer survived much longer and about half the animals were still alive when the experiment was ended in accordance with the protocol at day 22 (Fig. 6c).

## Discussion

Structural studies demonstrated that the pro-peptide of EntV (EntV^136^) forms a clasping palm motif that cages the α7 helix in a manner that portends its importance. Indeed, informed by this data, the antifungal activity was isolated to this single α-helix and just 10-12 amino acids have full to partial activity in multiple in vitro, ex vivo, and animal virulence models. Excitingly, these peptides not only prevented the onset of infection but, in the oral candidiasis model, effectively treated an established infection. They also reduced adhesion in vitro and in intravenous catheters implanted into rats, which models a key route of infection for human patients. Finally, EntV and the 12mer displayed efficacy in a systematic mouse model that mimics deadly human bloodstream infections.

No apparent toxicity was seen in the mouse model, and our previous study observed no toxicity when EntV was incubated with human and mouse cells^14^. Furthermore, the 12mer had no toxicity when incubated with human blood cells (Supplementary Fig. 6a). Interestingly, these shorter peptides no longer have antibacterial activity as measured in the *L. sakei* assay (Supplementary Fig 2). Furthermore, despite its robust protection against infection, EntV is not toxic to *C. albicans*. Growth of *C. albicans* was unaffected when EntV was added to shaking cultures in concentrations as high as 30 μM^14^. Rather than being fungicidal or fungistatic, EntV acts as an “anti-virulence” factor, interfering with key fungal virulence traits, including adhesion, morphogenesis, and biofilm formation. These have been linked to pathogenesis in a variety of fungal species, as well as in bacterial and protozoal pathogens.

The bacteriocin activity of EntV is non-lytic and therefore unlikely to form pores^16^. All four α-helices of EntV were required for this activity against *L. sakei*, although other bacterial species may respond differently and further in vitro, and microbiome study are necessary to determine the spectrum of activity. In contrast, α7 is all that is required for antifungal activity. Further mutational analysis and combinatorial library screening of this peptide is underway to better understand the features necessary for activity. Additionally, how EntV and derivative peptides exert antifungal activity is actively under study and preliminary evidence suggests binding to the fungal cell surface, which would be consistent with its ability to inhibit adhesion and invasion. Because adhesion is important for many fungal pathogens, this gives some optimism that it might be active against a broad array of species beyond what was demonstrated here. Further studies will be needed to better define the mechanism, species range, and target of this intriguing peptide.

The potential of peptides to broaden the pool of antimicrobial agents has been increasingly recognized. Two of the three classes of current antifungals are modified peptides (the polyenes and echinocandins) and a variety of other peptides have been investigated for their antifungal activity^34^. Included are several mammalian antimicrobial peptides active against fungi, such as Histatin-5 and CRAMP/LL-37. In this vein, peptides derived from EntV are promising leads for further optimization and development.

## Methods

### Ethics statement

Vertebrate animal experiments were conducted under protocols approved by the Animal Welfare Committees of the University of Wisconsin (rat catheter, protocol number DA0031) or The University of Texas Health Science Center at Houston (mouse OPC and mouse IV, protocol numbers AWC-18-0078 and AWC 21-0047) according to the guidelines of the Animal Welfare Act, The Institute of Laboratory Animals Resources Guide for the Care and Use of Laboratory Animals, and Public Health Service Policy. Animals were housed at 22 °C, ambient humidity, and with a 12 h light–dark cycle.

### Strains and media

Fungal strains were routinely propagated in YPD medium (1% yeast extract, 2% peptone, 2% dextrose). Most experiments used *C. albicans* wild-type strain SC5314^35^. For the adhesion assays the non-adherent strain HLC54 (*cph1∆::hisG/cph1∆::hisG efg1∆::hisG/efg1∆::hisG-URA3-hisG ura3/ura3*)^21^ was used as a negative control. *C. auris* strain AR-382 (CDC Antimicrobial Resistance Bank), *C. neoformans* strain 40-71 (a gift of L. Ostrosky) and *C. albicans* strains TFPY412 and TFPY1002 (gifts of T. Patterson and J. Lopez-Ribot^27^) were used in the assays shown in Fig. 4.

### Protein expression and purification

Strain CEGE1, a BL21 (DE3) derivative containing pET28a encoding EntV^136^ ^14^, was used for overexpression. 10 mL overnight culture was diluted into 1 L M9 SeMET (selenomethionine) media containing selection antibiotic ampicillin and grown at 37 °C with shaking till OD_600_ reached 1.2. The cell culture was induced with IPTG after switching temperature to 20 °C and left shaking overnight. Cell pellets were collected by centrifugation at 4735 xg, resuspended in binding buffer [100 mM HEPES pH 7.5, 300 mM NaCl, 5 mM imidazole and 5% glycerol (v/v)] and sonicated. Insoluble debris was removed by centrifugation at 22,617 xg. Ni-NTA affinity chromatography was used for protein purification. The supernatant was loaded on a column with 4 mL Ni-NTA resin (QIAGEN), pre-equilibrated with binding buffer with 0.5 mM TCEP, washed with 120 mL washing buffer [100 mM HEPES pH 7.5, 300 mM NaCl, 30 mM imidazole, 5% glycerol (v/v) and 0.5 mM TCEP], and protein was eluted with elution buffer [100 mM HEPES pH 7.5, 300 mM NaCl, 250 mM imidazole, 5% glycerol (v/v) and 0.5 mM TCEP]. The His-tag was cleaved by adding human thrombin (5 units per mg of recombinant protein) and 2.5 mM calcium chloride, in overnight dialysis against buffer, containing 20 mM Tris-HCl pH 8.0, 0.1 M NaCl, 0.5 mM TCEP and 5 mM Imidazol. To remove the His-tag, this was applied to a second Ni-NTA column and the flow-through was collected, then the tag-free protein was dialysed against crystallization buffer (10 mM HEPES pH 7.5, 300 mM NaCl), and the purity of the protein was analyzed by SDS-polyacrylamide gel electrophoresis.

### Crystallization and structure determination

The EntV^136^ crystal was grown at room temperature using the vapor diffusion sitting drop method using 2 µL of a 25 mg/mL protein solution, 0.1 M Tris pH 8 and 28% (w/v) PEG4K. The crystal was cryoprotected with paratone-N oil prior to flash freezing in liquid nitrogen. Diffraction data at 100 K at beamline 19-ID of the Structural Biology Center of the Advanced Photon Source, Argonne National Laboratory. HKL3000^36^ was used to process two Se-Met diffraction data sets. Computational corrections for absorption in a crystal and Lorentz factor were applied^19,20^. Anisotropic diffraction correction was necessary for structure solution^19,37,38^. Indexing, integration, and scaling indicated C2 symmetry. The best Se-Met crystal, used in the refinement, diffracted to a nominal resolution of ~1.8 Å but diffraction was highly anisotropic, with diffraction pattern extending to ~1.9 Å resolution in the *a* direction (<I>/<σ(I)> ~2) and to a resolution much higher than 1.8 Å in the *b* and *c* directions (<I>/<σ(I)> ~19 and ~13, respectively). Initial phases were obtained from a single Se-Met crystal in a single-wavelength anomalous diffraction (SAD) experiment by performing heavy atom search to resolution 2.2 Å using SHELXD^39^. The search identified 2 Se positions with correlations coefficients: CC_All_ = 31.3%, CC_Weak_ = 25.9%, and C_FOM_ = 57.3% with relative occupancies of 1.000 and 0.660. The handedness of the best solution was determined with SHELXE. The heavy atom positions were refined to 1.8 Å with MLPHARE^39^, with the final FOM reaching 0.185 for all observations. Density modification was performed with DM^40–42^. The resulting electron density map was used as the entry model for the model building with BUCCANEER^43^ and refinement with REFMAC^44^, run within HKL3000. The resulting main chain was ~82% complete (116 aa) with ~79% of side chains docked into electron density maps (112 aa), and R factor = 23.3% and R_free_ = 24.3%. The resulting assembly was used to perform isomorphous replacement with another data set using MOLREP (^45^ run within HKL3000, and rebuilt and refined again with BUCCANEER and REFMAC run within HKL3000. The resulting main chain was ~90% complete (127 aa) with ~80% of side chains docked into electron density maps (113 aa), and R factor = 23.4% and Rfree = 27.7%. Refinement was completed using Phenix.refine^46^ and Coot^47^. *B*-factors were refined as anisotropic for protein atoms and isotropic for non-protein atoms and TLS parameterization was included in the refinement. Average *B*-factor and bond angle/length RMSD values were calculated using Phenix. All geometry was verified using the Phenix, Coot and wwPDB validation tools. The X-ray crystallographic statistics are in Supplementary Table 1 and the structure was deposited to the Protein Data Bank under the accession code 7ROA.

### Peptide synthesis and alkylation

Peptides were synthesized and verified by Bio-Synthesis Inc (Lewisville, TX) or prepared in-house using standard Solid Phase Peptide Synthesis (SPPS) protocols. Starting from the C-terminal residue, the peptides were synthesized on Tentagel S-Ram 0.2–0.8 meq/g beads (from Chem Impex). FMOC protected peptide monomers (from Advanced ChemTech) were dissolved in dimethylformamide (DMF) at roughly a 3x molar excess (0.528 mM) relative to the manufacturer’s stated loading capacity (0.22 mM). The reactions were catalyzed by the addition of Hexafluorophosphate Benzotriazole Tetramethyl Uronium (HBTU) (200.11 mg per residue addition), Hydroxybenzotriazole (HOBt) (71.28 mg per residue addition), and N, N-Diisopropylethylamine (DIPEA) (68 µL per residue addition). Reaction completion for each residue was confirmed by conducting a ninhydrin test on a small sample of beads taken from the reaction vessel after washing the beads with DMF three times to clean any residual or unreacted components. Following completion of amino acid addition, the beads underwent a final deprotection to remove the FMOC group and then were washed with dichloromethane (DCM). Acid cleavage from the beads was accomplished by using Reagent B (88% v/v trifluoroacetic acid, 5% v/v phenol, 5% v/v ddH2O, 2% v/v triisopropylsilane). The volume of cleavage solution used was roughly 6 ml per reaction vessel. Synthesis quality was verified by performing HPLC and mass spectrometry. Prior to analysis, the peptides were ether precipitated for purification, dissolved in DMSO, and lyophilized. Yields of purified peptides, as a result of synthesis, ranged from 15 to 30 mg total. Peptide alkylation was conducted by reacting at least a 5x molar excess of iodoacetamide (0.4 M) to the wild type 12mer variant of EntV (1000 µM) for 48 h. The reaction was confirmed by monitoring the retention times of the peptides via HPLC. The product was then pooled by HPLC fraction collection and then dried down for use once the product weight was confirmed by mass spectrometry. All in-house and Bio-Synthesis verification of the peptides by HPLC and mass spectrometry is presented in Supplementary Data 2. The biophysical characteristics of the peptides are presented in Supplementary Table 2.

### *C. elegan*s killing assay

*C. elegans* infection assays were performed^13,14,48,49^. *C. elegans glp-4(bn2); sek-1(km-4)*^48^ nematodes were propagated and maintained on *E. coli* strain OP50^50^ that was seeded onto nematode growth medium (NGM) agar using standard techniques (Hope, 1999). To synchronize the animals, L1 stage worms on non-starved plates were washed off, filtered through a 10 μm filter (pluriSelect, pluriStrainer 10 μm), harvested by centrifugation at 750 xg for 30 seconds, transferred to OP50 seeded plates, and grown to the L4 stage. To prepare the infection plates, Fungal strains were grown in YPD broth for 24 h at 37 °C with agitation. 500 μl of the culture was plated onto BHI solid medium containing gentamycin (10 μg/ml) and grown for 24 h at 37 °C. The synchronized, L4 *C. elegans* were washed off the OP50 plates in 2 ml sterile M9 buffer and washed once, centrifuging at 750 xg for 30 seconds to collect animals. Animals were infected by placing them on the fungal lawn for 4 h at 25 °C. Following this exposure, they were washed off the plate and washed four times with 2 ml of sterile M9. The nematodes were then pipetted (~30 per well with two wells per condition for a total of ~60 worms assayed) into six-well plates with 2 ml of liquid medium (20% BHI broth and 80% M9) containing the indicated concentrations of test compounds. Plates were incubated at 25 °C, and worm death was scored daily. Kaplan–Meier survival curves were generated and analyzed as described in the quantification and statistical analysis section.

### *C. albicans* adhesion assays

Similar to the methodology in^51^, *C. albicans* strains were grown in YPD broth for 18 h at 30 °C with agitation and then subcultured for 4 h in the same conditions. Cells were then collected by centrifugation at 16,000 xg for 30 ss, washed twice in PBS, and adjusted to a concentration of 1 × 10^7^ cells/ml (measured with Countess II, Life Technologies) in PBS treated with the indicated concentrations of test compounds. The cell suspension was incubated for 1 h at 30 °C with agitation. Then 100 µl of the cell suspension was added to wells of a 96 well tissue culture treated polystyrene plate (Falcon) in addition to 100 µl of artificial saliva media^14,52^ containing the indicated concentrations of test compounds. The plate was then incubated at 37 °C for 90 min.

After incubation, the media was gently removed along with any cells that failed to adhere. The adhered cell layer was then stained for 20 min with 40 µl of 0.08% crystal violet solution (diluted, Sigma). The crystal violet stain was removed, and the wells were then washed three times with sterile water. Adhered cells were then destained with 200 µl of 200 proof ethanol for 20 min, and 100 µl of the ethanol solution was transferred to a new well for analysis. The optical density at 595 nm (OD_595_) was measured using a Synergy H1 plate reader (BioTek) with Gen5 version 3.08 software (BioTek).

### Lactobacillus MIC assay

For this assay, *Lactobacillus sakei* ATCC 15521^53^ was used following the methodology as described with a few modifications^54^. Briefly *L. sakei* was grown in Lactobacilli MRS broth (Difco, Detroit, MI, USA) for 18 h at 30 °C without agitation^55^. Following adjustment to a concentration of 1 × 10^6^ cells/ml, cells were diluted 1:10 into fresh MRS medium containing the indicated concentrations of EntV fragments. After 24 h of growth at 30 °C without agitation, OD_625_ readings were taken using a BioTek Cytation5 plate reader. Readings of blanks (containing fresh MRS medium) were subtracted from sample wells.

### Hemolysis assay

De-identified and pooled RBCs were purchased from Rockland Immunochemicals, Inc. RBCs were prepared at 6 × 10^7^ cells/mL in PBS. To 25 μL of peptide diluted appropriately to result in a final concentration of 1 nM and 1 µM, 125 μL of RBC suspension was added. The plates were gently rocked for 1 h at room temperature. The cells were then removed from suspension by centrifugation at 1000 xg for 5 min. From the experimental plate, 100 μL of the supernatant was carefully withdrawn from each well and added to a new 96-well plate. The absorbance of the solutions in each well was measured at 410 nm and each well was compared to a no-treatment control and a 100% lysis control (1% SDS).

### Peptide serum stability assay

One mL of RPMI supplemented with 10% (v/v) of human serum (type AB) were aliquoted and temperature-equilibrated at 37 °C for 15 min before adding a concentrated stock peptide solution resulting in a final peptide concentration of 100 µM. For each time point, 100 µL of the reaction solution was removed and added to 200 µL 96% ethanol for precipitation of serum proteins. The sample was cooled (4 °C) for 15 min and then spun at 18,000 g for 2 min to pellet the precipitated serum proteins. The reaction supernatant was analyzed using HPLC with absorbance detected at 280 nm. The peptide peak was detected first without serum treatment to assess the area under the curve for the peptide of interest. At each time point the area under the curve was calculated by HPLC and then divided by the untreated peptide area under the curve to determine the percent remaining.

### Mouse OPC model

The efficacy of EntV fragments were tested in the OPC model^14,56^. BALB/c mice, 10 wks, 18-20 g, were immunosuppressed by injecting 225 mg/kg cortisone acetate subcutaneously 1 d before inoculation, and subsequently on days 1 and 3 of the infection. To prepare the inoculum, 1 ml of *C. albicans* (SC5314) overnight culture grown at 30 °C in YPD broth was washed twice in PBS before resuspension in sterile Hanks’ balanced salt solution (HBSS) at a concentration of 1 × 10^6^ cells/mL. Calcium alginate swabs were soaked in this inoculum for 5 min prior to inoculation. Mice were anesthetized using ketamine and xylazine and placed on pre-warmed isothermal pads and the swabs were placed sublingually for 75 min. Mice were given additional doses of ketamine (50 mg/kg), as necessary. After inoculation, mice were given drinking water with EntV fragments or DMSO (0.01%) in the drinking water ad libitum. Mice were euthanized at 5 d after inoculation, unless noted. The tongues were excised and cut in half laterally for tissue histology and assessment of fungal burden.

For tissue histology the tongue sample was placed in 10% zinc-buffered formalin overnight and stored in 80% ethanol, before embedding in paraffin. For each tongue, 5 μm sections were prepared using a Leica microtome and stained using Periodic Acid-Schiff (PAS) stain. Epithelial invasion was measured from 40x images taken of the entire tissue section of each tongue half with the total area of epithelium and infected epithelium were measured using ImageJ (version 1.6). Measurements were totaled and expressed as a percentage of total infected epithelium relative to the entire epithelial area^14,57^.

For examination of fungal burden, qPCR was used by amplifying a 269-bp fragment of the internal transcribed spacer 2 (ITS2) between the 5.8 S and 28 S ribosomal RNA genes of *C. albicans* using oligonucleotides GTGAATCATCGARTCTTTGAAC and TATGCTTAAGTTCAGCGGGTA, as the forward and reverse primers, respectively^14,58^. DNA was extracted from homogenized tongue tissue using the Yeast DNA Extraction Kit (Thermo Scientific) according to the protocol with minor modifications. The ITS2 fragment was amplified by qPCR with a CFX96 Real-Time System with a C1000 Touch thermal cycler (BioRad). *C. albicans* gDNA was quantified using FastStart Universal SYBR Green master mix with ROX (Roche). To screen for contamination and background fluorescence during the qPCR amplification, no template controls were used.

### Intravenous infection model

The disseminated candidiasis model^59,60^ was performed by growing *C. albicans* SC5314 overnight in YPD at 30 °C, then diluting 1/100 and growing in a second overnight. The late-log phase culture was collected by centrifugation at 16,000 xg for 30 s, washed in PBS, and diluted to 5 × 10^6^ cells/ml in PBS containing vehicle (0.01% DMSO) or 100 nM EntV or the 12mer peptide and incubated for 2 h at room temperature. Female ICR mice 6 wks, 18–20 g, 10/experimental group were inoculated via the lateral tail vein with 0.1 ml. Animals were monitored at least twice daily for 21 days for signs of moribundity.

### Rat catheter model

Indwelling central venous catheters were implanted in the jugular veins of 400 g, 16 wk old, female Sprague-Dawley rats and were inoculated with 1 × 10^6^ cells/mL *C. albicans* with or without 100 nM EntV or 20 nM 12-mer peptide (group size was 3)^33^. To quantitate fungal burden after 24 h, the catheter was removed, and 1 cm of the tip placed in a one milliliter of sterile 0.9% NaCl followed by vortexing. A 1:10 serial dilution of this wash fluid was plated on SDA and colonies counted after 24 h of growth at 35 °C. For scanning electron microscopy (SEM), catheters were fixed overnight in 4% formaldehyde and 1% glutaraldehyde in PBS. The catheters were then washed with PBS and treated with 1% osmium tetroxide in PBS for 30 min. Serial ethanol washes and critical point drying were used to dry the segments before they were mounted, and palladium-gold coated. All images were taken using a SEM LEO 1530, with Adobe Photoshop CC (20.0.4 release) used for image compilation.

### Quantification and statistical analysis

GraphPad Prism 9.0 was used for all data analysis. For the fungal adhesion, bacterial MIC, and hemolysis assays, means of the experimental conditions were calculated and compared to the DMSO condition. Lines with error bars indicate the mean and the standard deviation (SD). Significance was determined using one-way ANOVA followed by Dunnett’s multiple comparison test. For the OPC fungal burden and % hyphal invasion measurements, means of the experimental conditions were calculated and compared to other conditions as indicated in the individual panels. Lines with error bars indicate the mean and the standard error of the mean (SEM). Significance was determined using one-way ANOVA followed by Tukey’s multiple comparison test. For the catheter experiments, the means and the standard deviations were calculated, and an unpaired, two-tailed *t* test was used to compare the treatments to the controls. Mantel–Cox log rank analysis was used to compare survival curves. The median survival and comparison values for all *C. elegans* survival experiments and their replicates can be found in Supplementary Data 1. For all statistical tests, *p* values <0.05 were considered statistically significant and asterisks in the figure panels indicate the levels of significance as follows: *p < 0.05, **p < 0.01, ***p < 0.001, ****p < 0.0001.

### Reporting summary

Further information on research design is available in the Nature Research Reporting Summary linked to this article.

## Supplementary information

Supplementary Information

Description of Additional Supplementary Files

Supplementary Data 1

Supplementary Data 2

Reporting Summary

## Data Availability

All data generated in this study are available in the main text, supplementary materials, or the source data file except for the structural data that was deposited to the Protein Data Bank under the accession code 7ROA. Source data are provided with this paper.

## Source data

Source Data
