# Supplementary material for: Structural and functional analysis of EntV reveals a 12 amino acid fragment protective against fungal infections
Source: Nat Commun. 2022 Oct 13;13:6047. doi: 10.1038/s41467-022-33613-1 (PMC9562342; doi:10.1038/s41467-022-33613-1)
Supplement: Supplementary file 1 — Supplementary Information [file 41467_2022_33613_MOESM1_ESM.docx]

**SUPPLEMENTARY INFORMATION**

**Supplementary Table 1. X-ray crystallographic statistics.**

| **PDB code** | 7ROA |
| --- | --- |
| **Data collection** |  |
| Space group | C2 |
| Unit cell  *a*, *b, c*, Å  α, β, γ, ° | 62.39, 32.06, 49.99  90, 90.86, 90 |
| Resolution, Å | 50.00 – 1.80 |
| *R*_merge_^a^  *R*_pim_^b^ | 0.138 (0.671)*  0.055 (0.290) |
| *CC*_1/2_ | 0.913 (0.618) |
| *I* / σ(*I)* | 14.2 (6.9) |
| Completeness, % | 98.6 (99.1) |
| Redundancy | 6.2 (5.2) |
|  |  |
| **Refinement** |  |
| Resolution, Å | 31.2 – 1.82 |
| No. unique reflections:  working, test | 8823, 685 |
| *R*-factor/free *R­*-factor^c^ | 17.7/17.5 (20.5/26.9) |
| No. refined atoms  Protein  Water | 904  87 |
| *B*-factors  Protein  Water | 24.6  39.5 |
| r.m.s.d.  Bond lengths, Å  Bond angles, ° | 0.006  0.858 |

*values in brackets refer to highest resolution shells.

^a^*R*_merge_ = Σ_hkl_Σ_j_|*I*_hkl.j_ - 〈*I*_hkl_〉|/Σ_hkl_Σ_j_I_hk,j_, where *I*_hkl,j_ and 〈*I*_hkl_〉 are the *j*th and mean measurement of the intensity of reflection *j*.

^b^*R*_pim_ = Σ_hkl_√(n/n-1) Σ^n^_j=1_|*I*_hkl.j_ - 〈*I*_hkl_〉|/Σ_hkl_Σ_j_*I*_hk,j_

^c^*R* = Σ|F_p_^obs^ – F_p_^calc^|/ΣF_p_^obs^, where F_p_^obs^ and F_p_^calc^ are the observed and calculated structure factor amplitudes, respectively.

**Supplementary Table 2. Biophysical characteristics of peptides.**

| **Peptide name** | **Expected MW** | **Observed MW** | **Solubility** |
| --- | --- | --- | --- |
| EntV | 7217.70 | 7217.57 | 10 µM + |
| α4 | 1513.79 | 1513.68 | 10 µM + |
| α5 | 1427.76 | 1426.39 | 10 µM + |
| α6 | 2115.56 | 2116.29 | 10 µM + |
| α7 | 2028.41 | 2028.56 | 10 µM + |
| α4-6 | 4621.57 | 4620.61 | 10 µM + |
| α7-random | 2028.41 | 2027.90 | 10 µM + |
| α7-disrupted | 2028.41 | 2028.40 | 10 µM + |
| 16aa | 1612.97 | 1612.03 | 10 µM + |
| 14aa-I | 1484.83 | 1484.27 | 10 µM + |
| 14aa-II | 1452.77 | 1452.98 | 10 µM + |
| 13aa | 1371.67 | 1372.20 | 10 µM + |
| 12aa | 1258.51 | 1258.08 | 10 µM + |
| 11aa | 1159.38 | 1159.59 | 10 µM + |
| 10aa | 1088.30 | 1088.49 | 10 µM + |
| 9aa | 1031.25 | 1031.66 | 10 µM + |
| α7-EE | 1614.94 | 1614.68 | 10 µM + |
| α7-II | 1583.02 | 1583.76 | 10 µM + |
| C->S | 1241.67 | 1240.74 | 10 µM + |
| ALK | 1302.68 | 1303.66 | 10 µM + |

**Supplementary Fig. 1. The charged residues of EntV are located asymmetrically. a** The outer surface of the N-terminal domain (NTD) of EntV^136^ is negatively charged (red) whereas the outer surface of the C-terminal domain (CTD) is positively charged (blue). **b** The structure of EntV^68^ (α4- α7) modeled by removal of the NTD. The outer surface of α4- α6 is positively charged whereas the inner surface that largely faces α7 is hydrophobic/neutral.

**Supplementary Fig. 2. EntV fragments have no inhibitory activity against *Lactobacillus sakei.*** *L. sakei* was cultured in Lactobacilli MRS broth overnight and diluted 1/10 into fresh medium to which EntV and EntV fragments were added at various concentrations. Following 24 hours of growth at 30°C, the OD_625_ readings were taken. The mean of three biological replicates were measured and the error bars indicate the SD. Significance compared to the DMSO control for all conditions was determined using one-way ANOVA followed by Dunnett’s multiple comparison test. Only those that were statistically significant are marked and all had a p < 0.0001.

**Supplementary Fig. 3. Some substitutions of the charged residues of α7 affect antifungal activity. a** and **c** show survival over time of *C. elegans* infected with *C. albicans* and exposed to 1nM of the indicated peptides. The glutamines were changed to glutamates in α7-EE and to isoleucines in a7-II in **a**. The cysteine of the 12mer was changed to a serine or chemically alkylated in **c**. Asterisks indicate the degree of statistical difference in survival compared to animals exposed to EntV^68^ following Mantel-Cox log rank analysis. An n of 60 was used and one representative trial is shown. Exact p values from top to bottom: **a** <0.0001, 0.9407, 0.0007, 0.8200. **c** <0.0001, <0.0001, and 0.3551. Median survival and p values of all trials are shown in Supplemental Data 1. **b** Adhesion of *C. albicans* to tissue-culture treated plates following incubation with indicated concentrations of α7 fragments. “Neg” is a control non-adherent strain (∆*efg1 ∆cph1*). Lines with error bars indicate the mean and the SD following normalization against the mean of the vehicle control group. Significance was determined using one-way ANOVA followed by Dunnett’s multiple comparison test. Lines with error bars indicate the mean and the SD following normalization against the mean of the vehicle control group. Statistical significance in comparison to the DMSO control group was determined using one-way ANOVA followed by Dunnett’s multiple comparison test. An n of four biological replicates, each with six technical replicates, was used. Exact p values from left to right are as follows: <0.0001, 0.7944, <0.0001, 0.9994, 0.0441, 0.9995, 0.7324, 0.9994, 0.3030, 0.9876.

**Supplementary Fig. 4. Shorter variants of α7 retain adhesion activity down to 10aa.** Adhesion of *C. albicans* to tissue-culture treated plates following incubation with the indicated concentrations of EntV **a** and α7 variants of 16aa **b**, 12aa **c**, 11aa **d**, 10aa **e** and 9aa **f**. “Neg” is a control non-adherent strain (∆*efg1* ∆*cph1*). Lines with error bars indicate the mean and the SD following normalization against the mean of the vehicle control group. Significance was determined using one-way ANOVA followed by Dunnett’s multiple comparison test. Lines with error bars indicate the mean and the SD following normalization against the mean of the vehicle control group. Statistical significance in comparison to the DMSO control group was determined using one-way ANOVA followed by Dunnett’s multiple comparison test. An n of three (**a-b**) or four (**c-f**) biological replicates, each with six technical replicates, was used. Exact p values from left to right are as follows: **a** 0.1407, 0.0170, 0.9998, 0.9997, 0.9999, 0.9998, 0.0009, **b** 0.0002, 0.0003, 0.8038, 0.9502, 0.7657, 0.0003, **c** <0.00001, <0.0001, 0.0004, 0.9973, 0.9998, 0.9999, <0.0001, **d** 0.0008, 0.0028, 0.9523, 0.9930, 0.9594, 0.9997, <0.0001, **e** <0.0001, <0.0001, 0.9336, 0.8043, 0.9999, 0.9998, <0.0001, **f** 0.9627, 009518, >0.9999, >0.9999, 0.9840, 0.9995, and <0.0001. **Supplementary Fig. 5. The 12mer is protective in a mouse model of oropharyngeal candidiasis.** **a** The percentage of the tongue surface showing hyphal invasion in animals treated with 100nM of different fragment lengths of the α7 peptide. **b** The percentage of the tongue surface showing hyphal invasion in animals at day 3 or 5 post-inoculation following treatment with 100nM of the 12mer starting at day 0 or 3. **c** The percentage of the tongue surface showing hyphal invasion in animals treated with different concentrations of the 12mer in comparison to fluconazole. Amount of *C. albicans* DNA detected by qPCR, **d**, and percentage of the tongue surface showing hyphal invasion, **e**, in animals treated with different concentrations of the 12mer. Amount of *C. albicans* DNA detected by qPCR, **f**, and percentage of the tongue surface showing hyphal invasion, **g**, disaggregated by sex in animals treated with 100nM of EntV. An n of 5-8 biologically independent animals was used with the exact number indicated by the number of data points. Averages were calculated and the error bars represent the SEM. Horizontal lines mark compared conditions and the exact p values are given. Significance was determined using one-way ANOVA followed by Tukey’s multiple comparison test. Exact p values from top to bottom are as follows: **a** 0.0003, 0.0019, 0.0065, >0.9999, **b** <0.0001, 0.0250, 0.0250, 0.0240, 0.9935, **c** 0.0304, <0.0001, <0.0001, 0.0002, 0.0228, >0.9999, 0.3923, 0.5257, 0.5011, **d** 0.0864, 0.0273, 0.9517, 0.0033, <0.0001, 0.0009, **e** 0.0152, 0.0383, 0.0015, 0.9663, 0.6514, 0.3885, **f** <0.0001, <0.0001, 0.0050, 0.0056, 0.1110, **g** <0.0001, <0.0001, 0.0135, 0.0011, 0.1550.

**Supplementary Fig. 6. Hemolytic and stability features of EntV and the 12mer**. **a** 1 nM and 1 µM of EntV or the 12mer were added to 6 x 10^7^ RBC cells/mL and percent lysis was determined following a 1-hour incubation. Using an n of 6 for the test samples and 3 for the 1% SDS control, the mean was calculated, and the error bars indicate the SD. Significance compared to the DMSO control was determined for all conditions using one-way ANOVA followed by Dunnett’s multiple comparison test. ****p < 0.0001. **b** The stability of 100 μM of EntV and 12mer in 10% human serum was determined over time. Using an n of 3, the mean of each time point was calculated, and error bars indicate the SEM.
