# Supplementary material for: Structural and functional analysis of EntV reveals a 12 amino acid fragment protective against fungal infections
Source: Nat Commun. 2022 Oct 13;13:6047. doi: 10.1038/s41467-022-33613-1 (PMC9562342; doi:10.1038/s41467-022-33613-1)
Supplement: Supplementary file 2 — Description of Additional Supplementary Files [file 41467_2022_33613_MOESM2_ESM.pdf]

## Description of Additional Supplementary Files

**Title**: Supplementary Data 1 (xlsx):

**Description**: C. elegans survival experiments' statistics and replicates III.

**Title:** Supplementary Data 2 (pdf):

**Description**: HPLC and mass spectrometry verification of all peptides
