# Supplementary material for: Structural and functional analysis of EntV reveals a 12 amino acid fragment protective against fungal infections
Source: Nat Commun. 2022 Oct 13;13:6047. doi: 10.1038/s41467-022-33613-1 (PMC9562342; doi:10.1038/s41467-022-33613-1)
Supplement: Supplementary file 4 — Supplementary Data 2 [file 41467_2022_33613_MOESM4_ESM.pdf]

sEntV

Column: 250X4.6mm 5u C18 120A
Solvent: A:0.05%TFA in Water; B: 0.05 % TFA in ACN
ID : CD-066/EQ-021

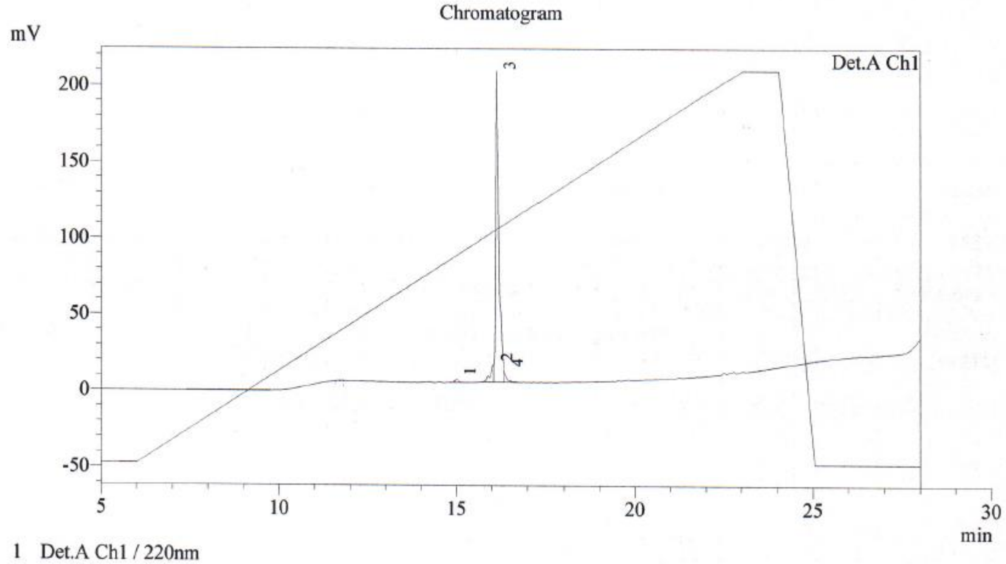

1 Det.A Ch1 / 220nm

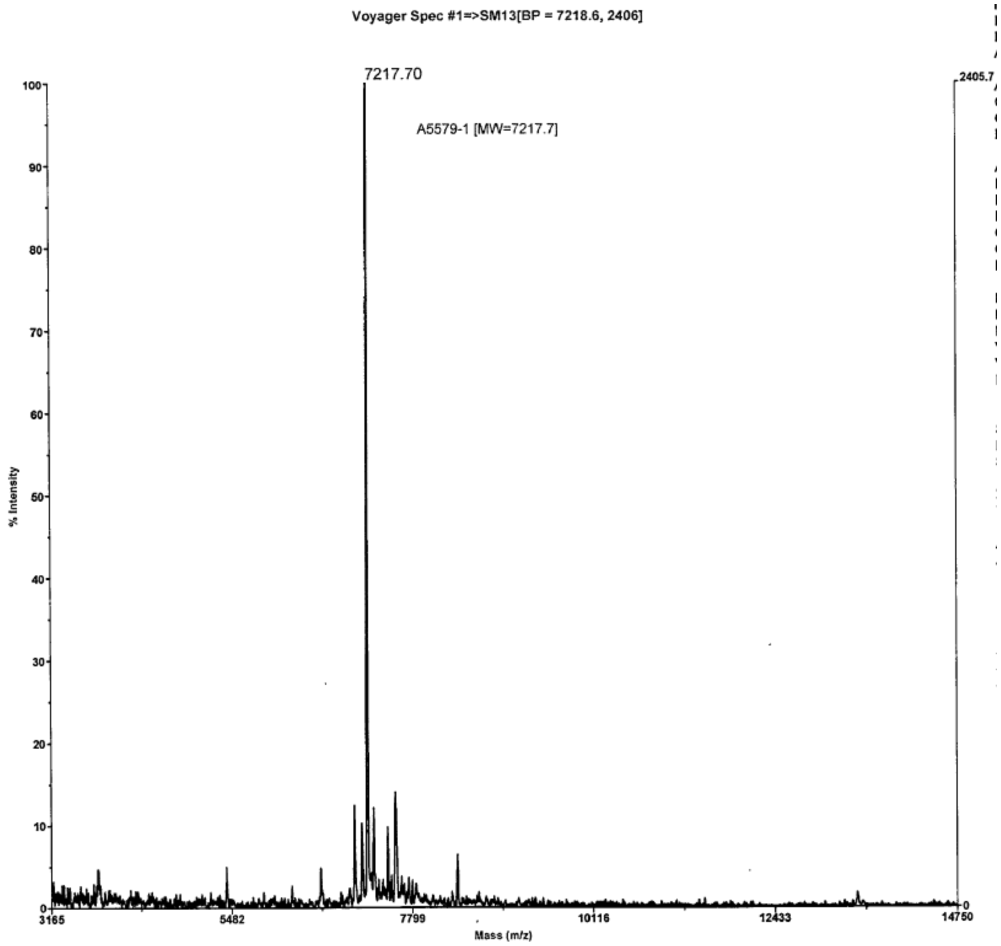

α4

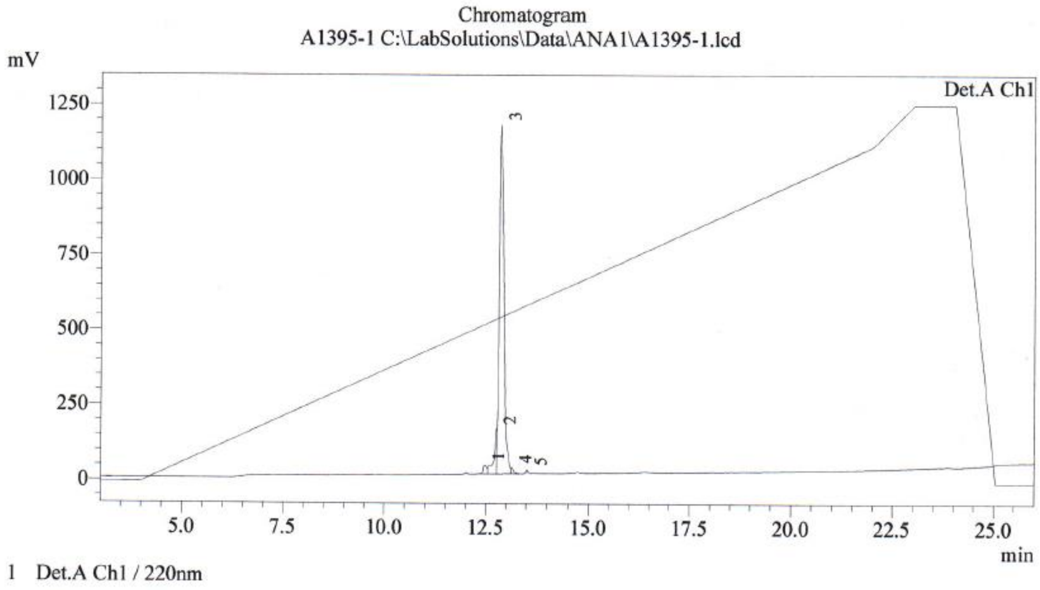

Chromatogram
A1395-1 C:\LabSolutions\Data\ANA1\A1395-1.lcd
mV
Det.A Ch1
1250
1000
750
500
250
0
5.0
7.5
10.0
12.5
15.0
17.5
20.0
22.5
25.0
min
1 Det.A Ch1 / 220nm

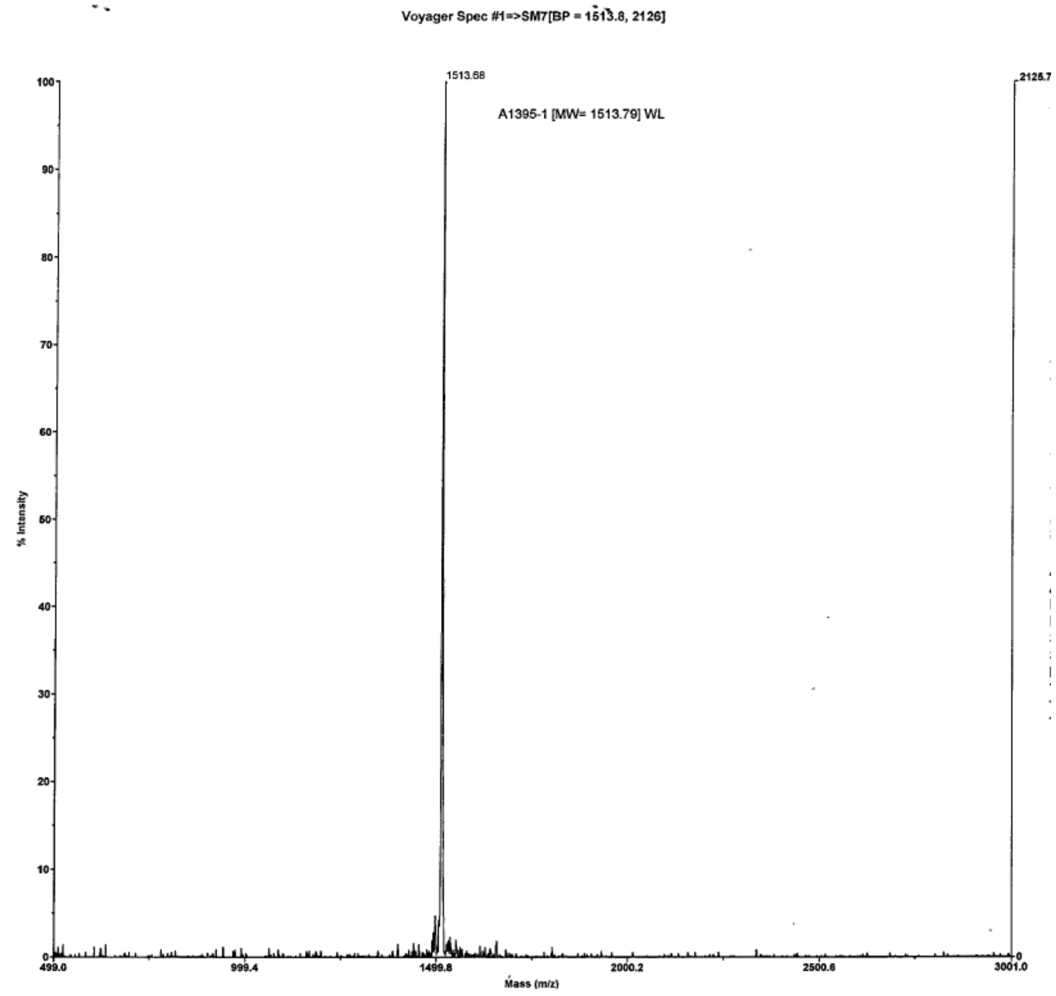

Voyager Spec #1=>SM7[BP = 1513.8, 2126]
1513.68
2125.7
A1395-1 [MW= 1513.79] WL
% Intensity
Mass (m/z)
499.0
999.4
1499.8
2000.2
2500.6
3001.0

α5

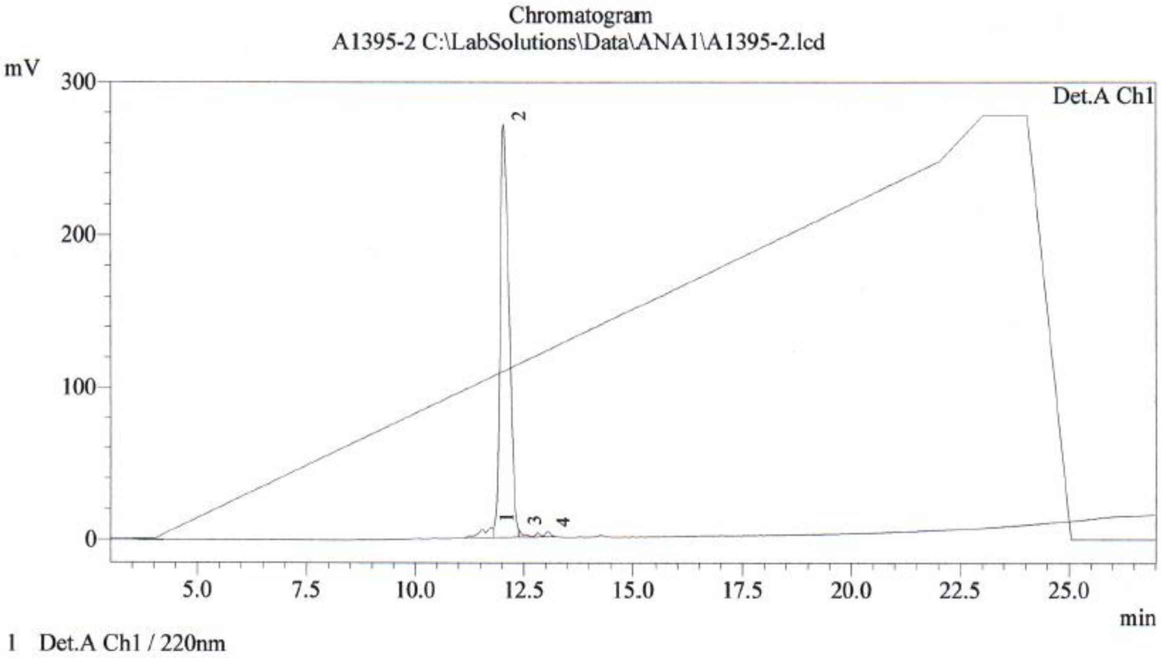

Chromatogram
A1395-2 C:\LabSolutions\Data\ANA1\A1395-2.lcd
mV
300
200
100
0
Det.A Ch1
2
1
3
4
5.0
7.5
10.0
12.5
15.0
17.5
20.0
22.5
25.0
min
1 Det.A Ch1 / 220nm

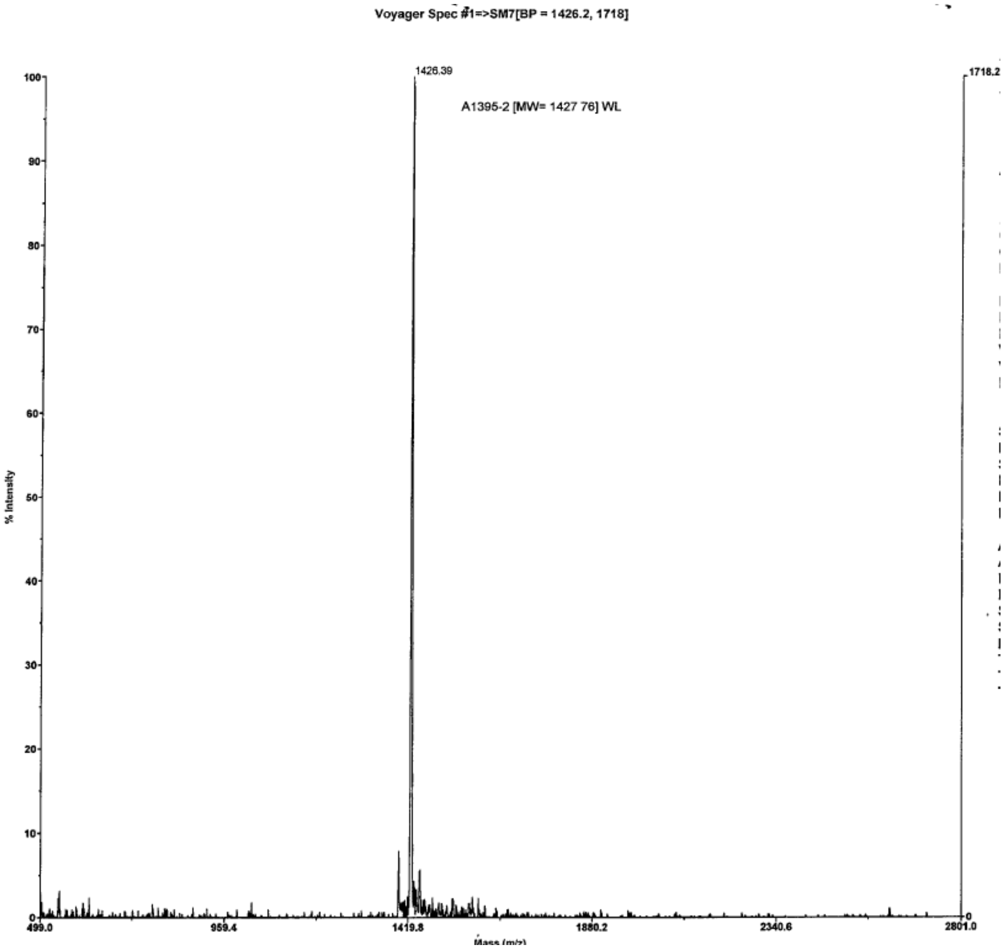

Voyager Spec #1=>SM7[BP = 1426.2, 1718]
1426.39
1718.2
A1395-2 [MW= 1427 76] WL
% Intensity
100
90
80
70
60
50
40
30
20
10
0
499.0
959.4
1419.8
1880.2
2340.6
2801.0

α6

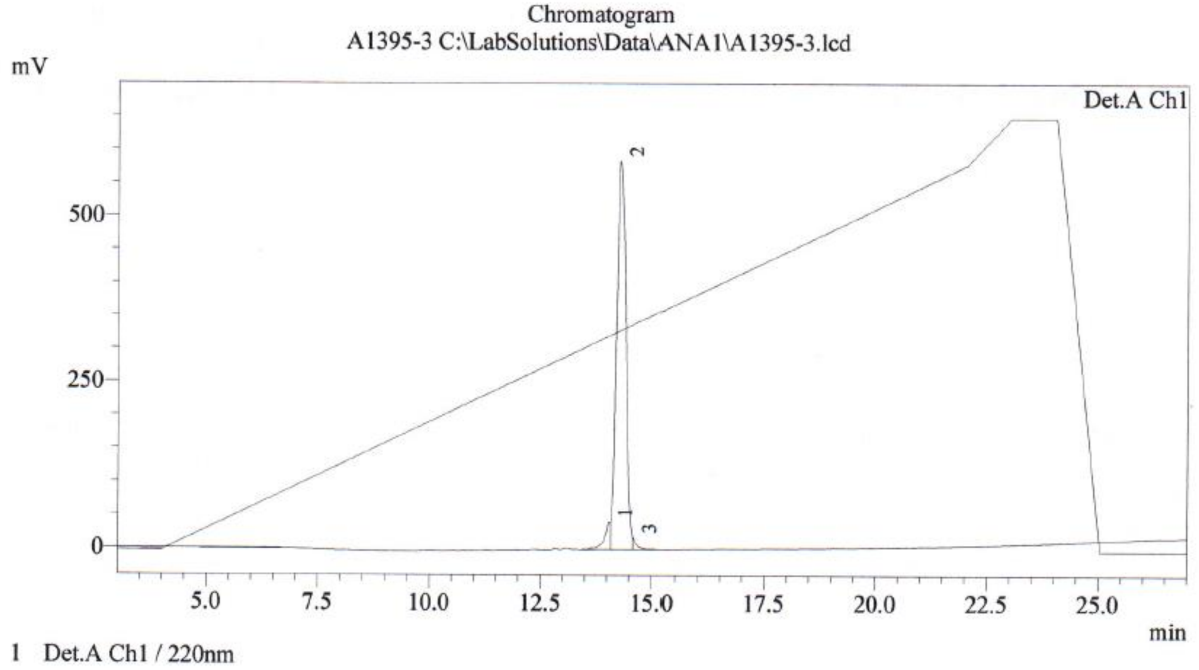
Chromatogram
A1395-3 C:\LabSolutions\Data\ANA1\A1395-3.lcd
mV
Det.A Ch1
500
250
0
5.0
7.5
10.0
12.5
15.0
17.5
20.0
22.5
25.0
min
1 Det.A Ch1 / 220nm

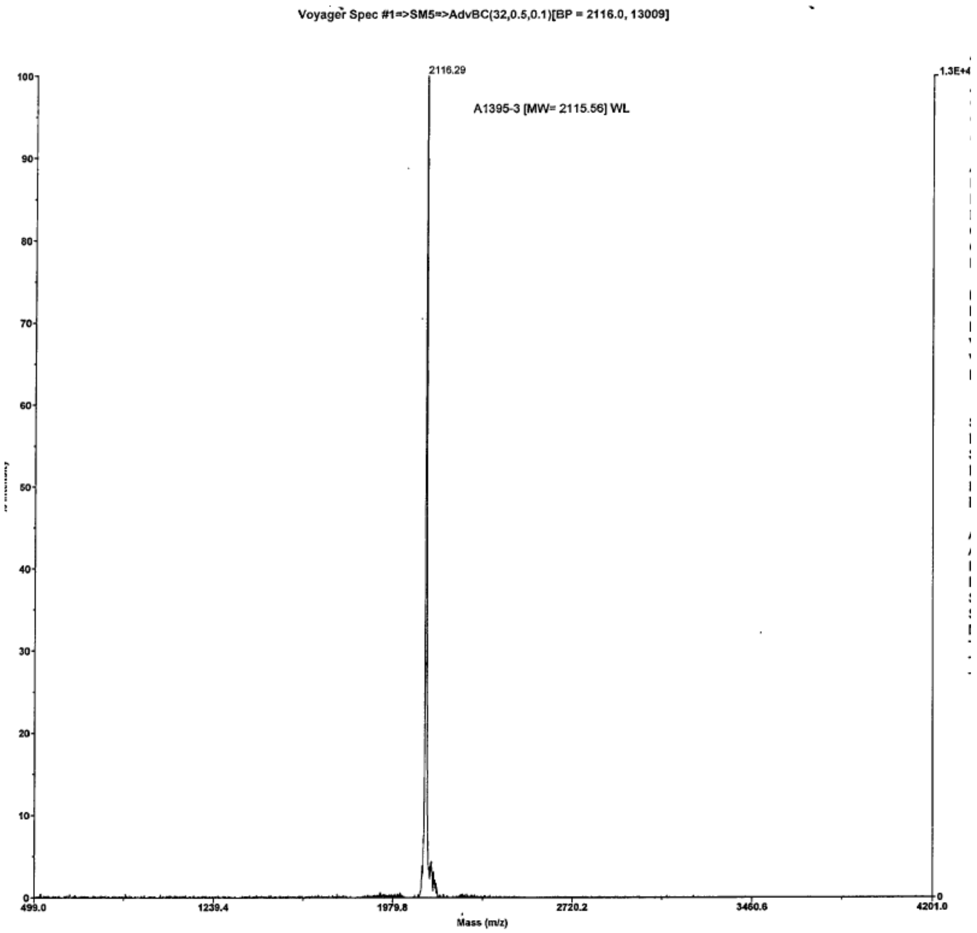
Voyager Spec #1=>SM5=>AdvBC(32,0.5,0.1)[BP = 2116.0, 13009]
2116.29
A1395-3 [MW= 2115.56] WL
1.3E+4
499.0
1239.4
1979.8
2720.2
3460.6
4201.0
Mass (m/z)

α7

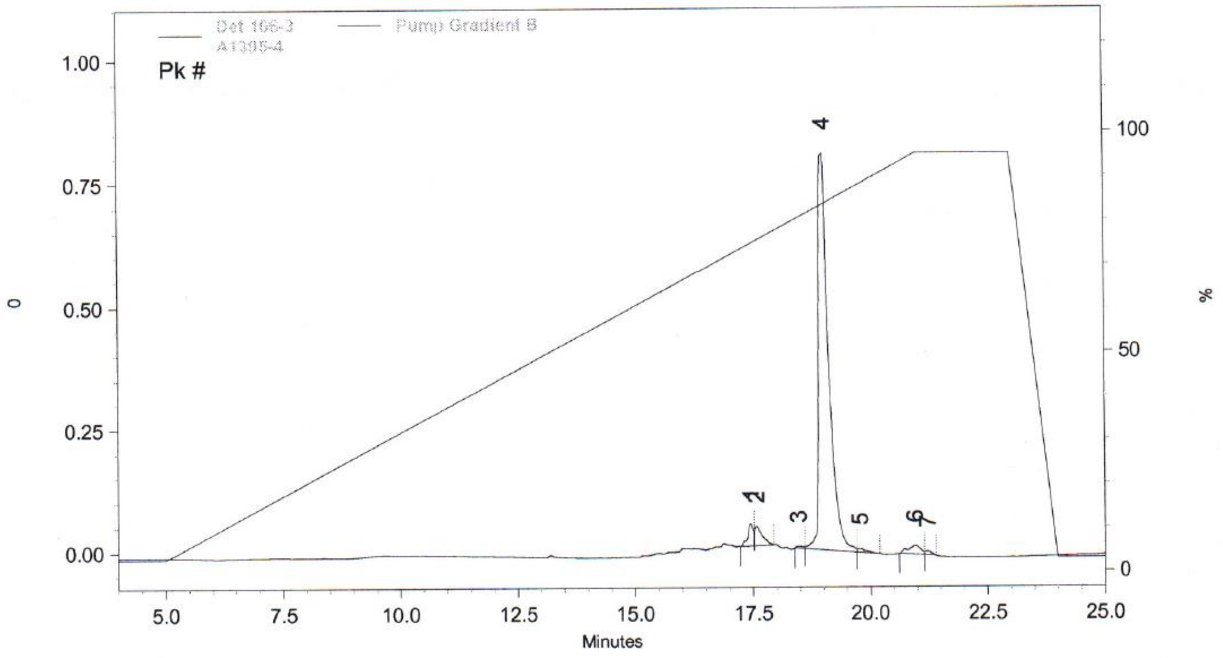
Det 166-3
A1395-4
Pump Gradient B
Pk #
1.00
0.75
0.50
0.25
0.00
0
%
100
50
0
1
2
3
4
5
6
7
5.0
7.5
10.0
12.5
15.0
17.5
20.0
22.5
25.0
Minutes

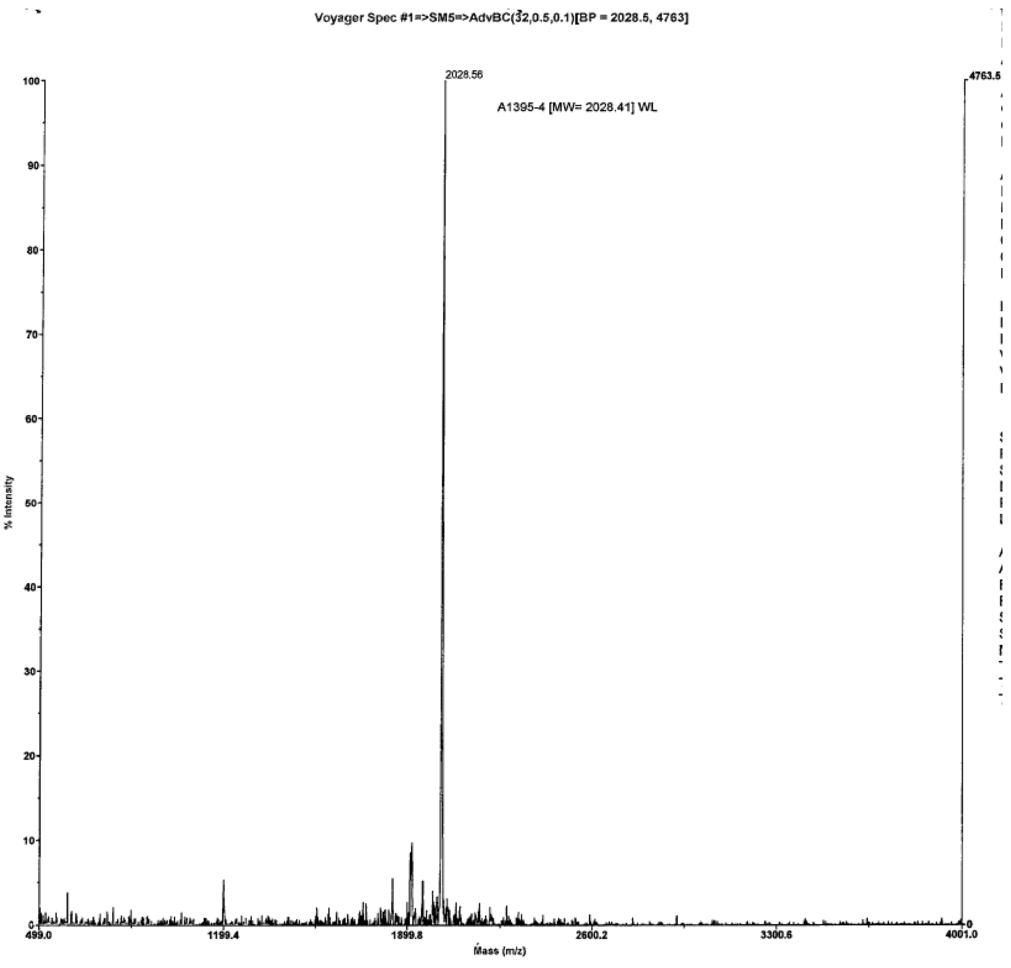
Voyager Spec #1=>SM5=>AdvBC(32,0.5,0.1)[BP = 2028.5, 4763]
2028.56
4763.5
A1395-4 [MW= 2028.41] WL
% Intensity
Mass (m/z)
499.0
1199.4
1899.8
2600.2
3300.6
4001.0

α4-6

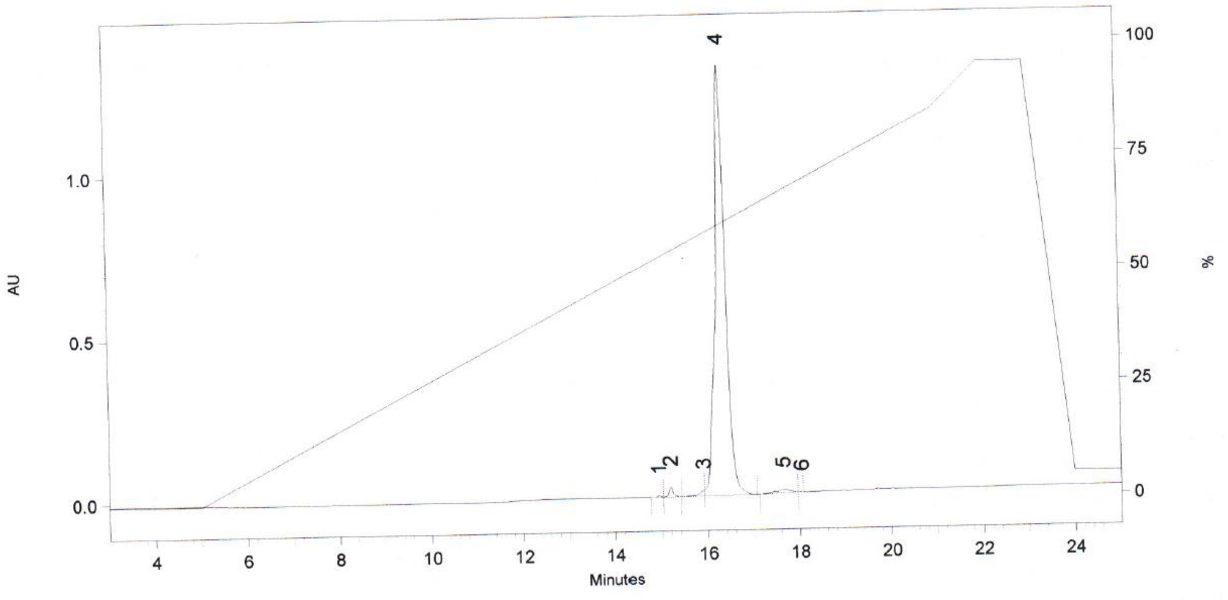

AU
%
Minutes
1
2
3
4
5
6

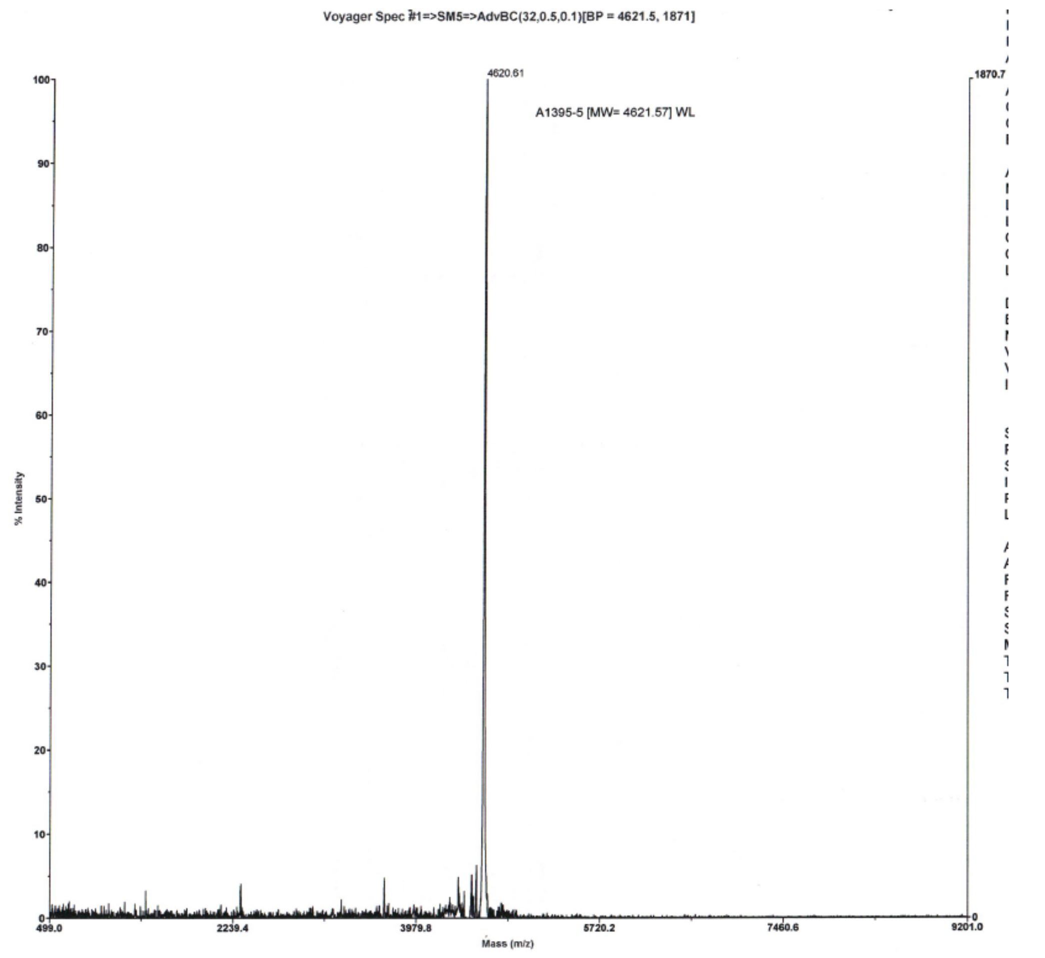

Voyager Spec #1=>SM5=>AdvBC(32,0.5,0.1)[BP = 4621.5, 1871]
4620.61
A1395-5 [MW= 4621.57] WL
1870.7
% Intensity
Mass (m/z)

α7-random

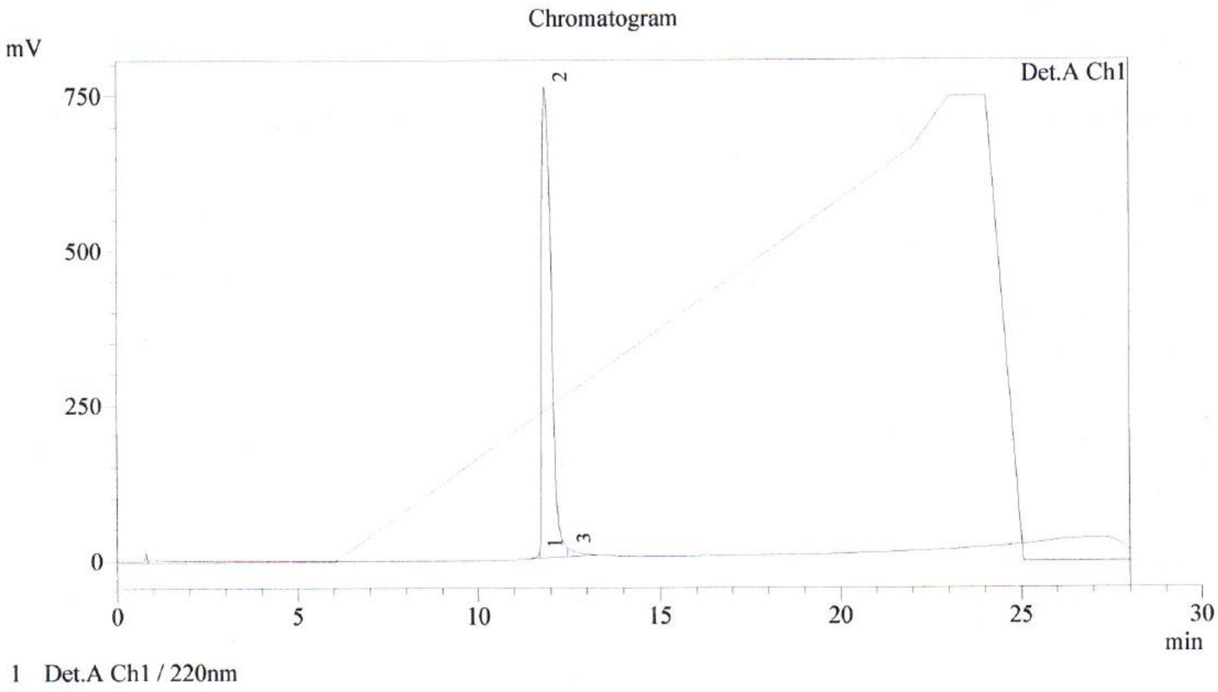
Chromatogram
mV
750
500
250
0
Det.A Ch1
2
1
3
0
5
10
15
20
25
30
min
1 Det.A Ch1 / 220nm

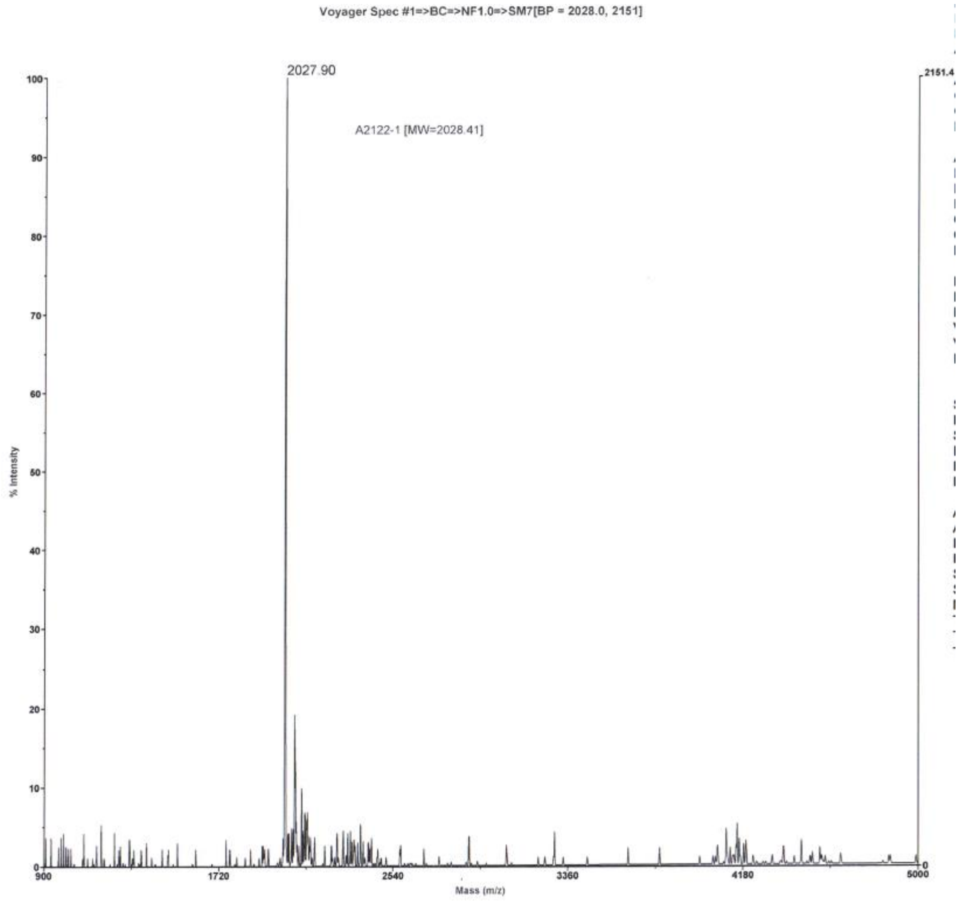
Voyager Spec #1=>BC=>NF1.0=>SM7[BP = 2028.0, 2151]
2027.90
2151.4
A2122-1 [MW=2028.41]
% Intensity
Mass (m/z)
900
1720
2540
3360
4180
5000

α7-disrupted

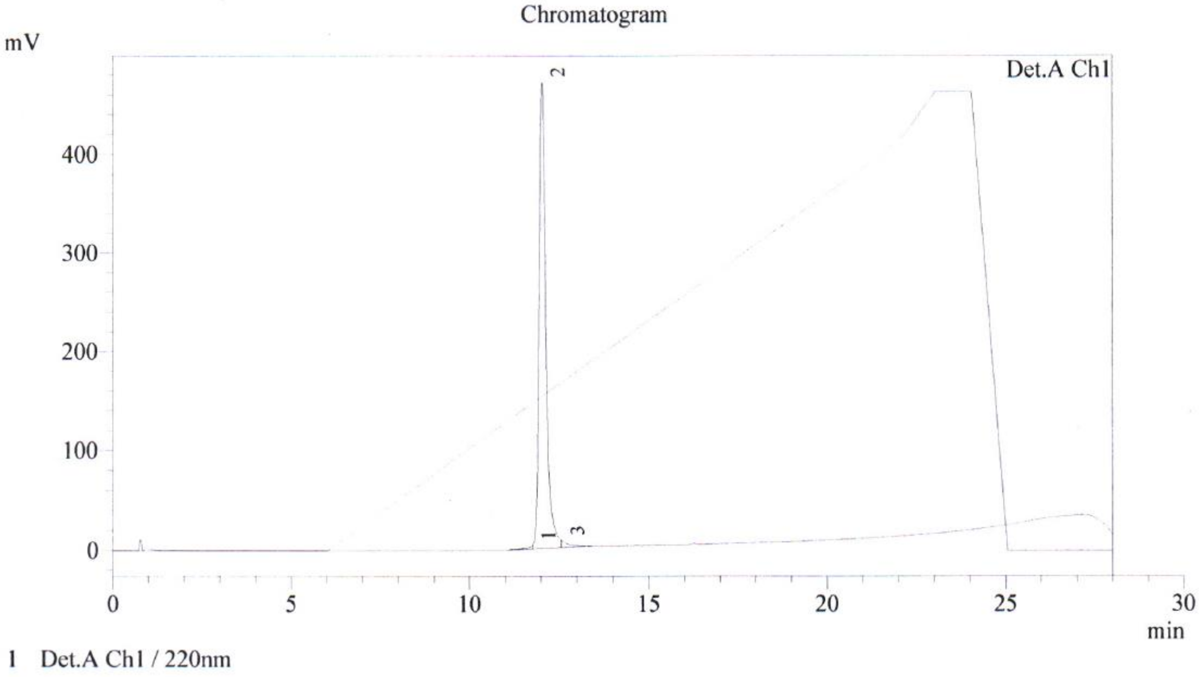

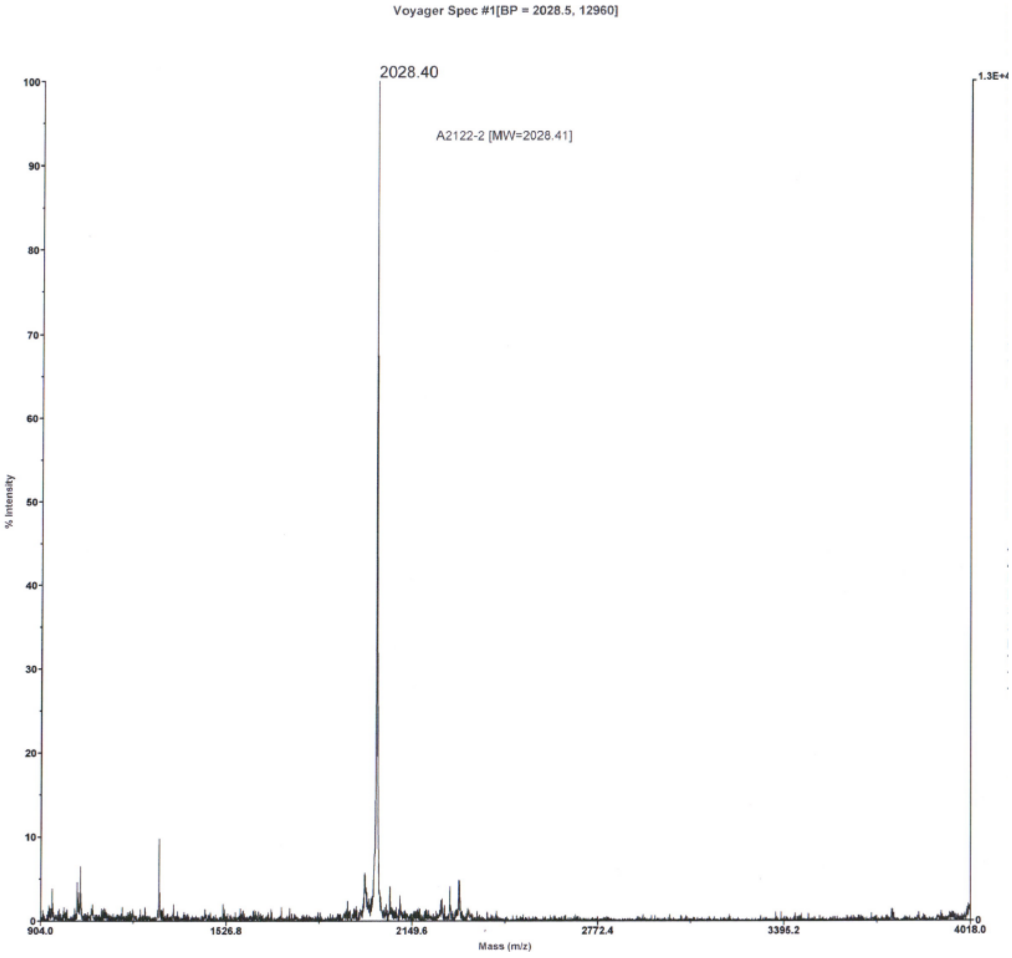

16aa

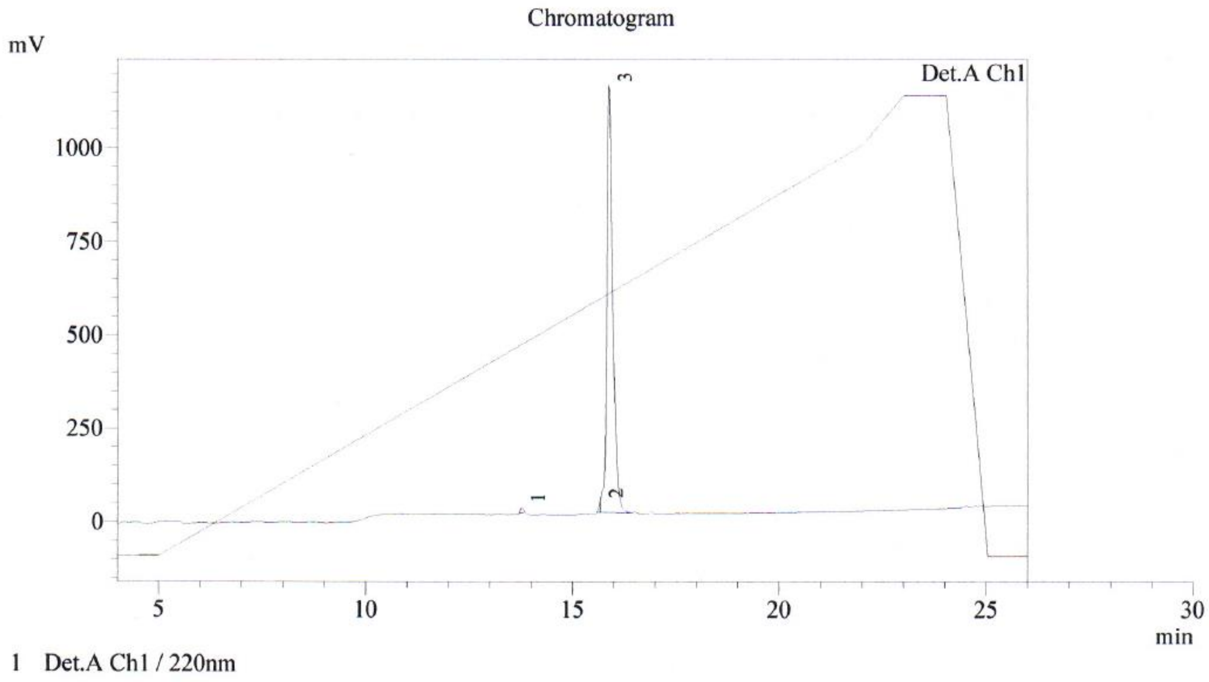
Chromatogram
mV
Det.A Ch1
1000
750
500
250
0
1
2
3
5
10
15
20
25
30
min
1 Det.A Ch1 / 220nm

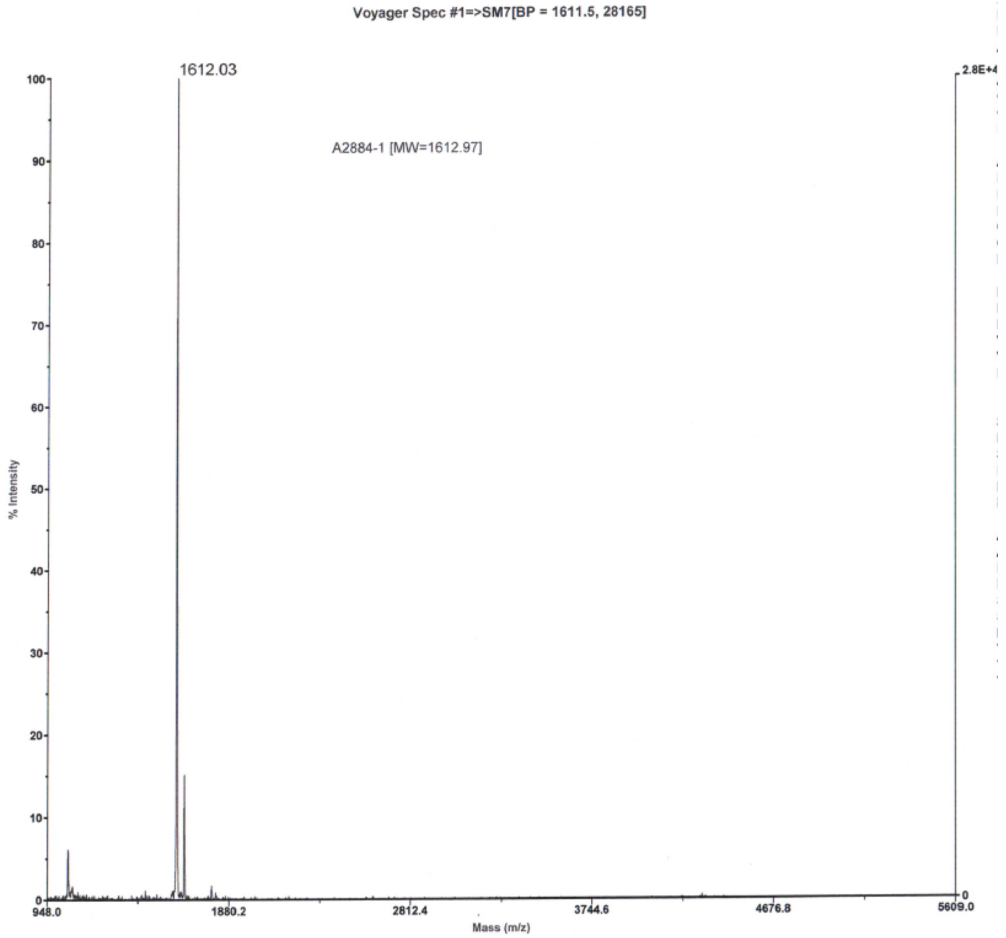
Voyager Spec #1=>SM7[BP = 1611.5, 28165]
1612.03
A2884-1 [MW=1612.97]
2.8E+4
% Intensity
100
90
80
70
60
50
40
30
20
10
0
948.0
1880.2
2812.4
3744.6
4676.8
5609.0
Mass (m/z)

14aa-I

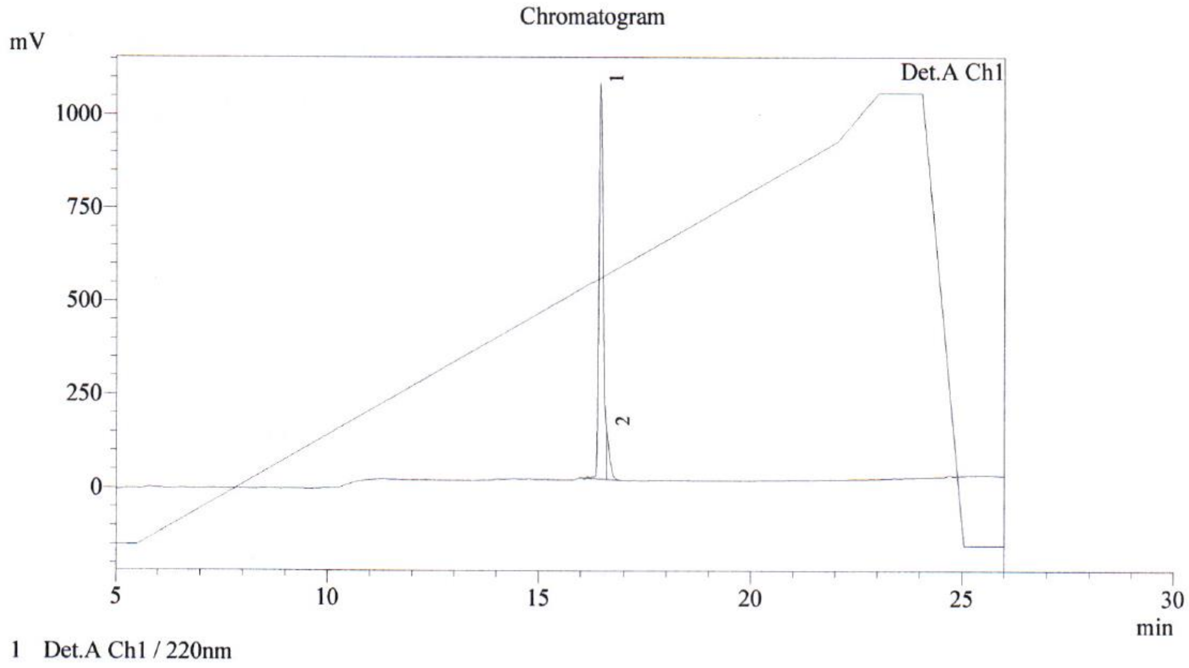
Chromatogram
mV
1000
750
500
250
0
Det.A Ch1
1
2
5
10
15
20
25
30
min
1 Det.A Ch1 / 220nm

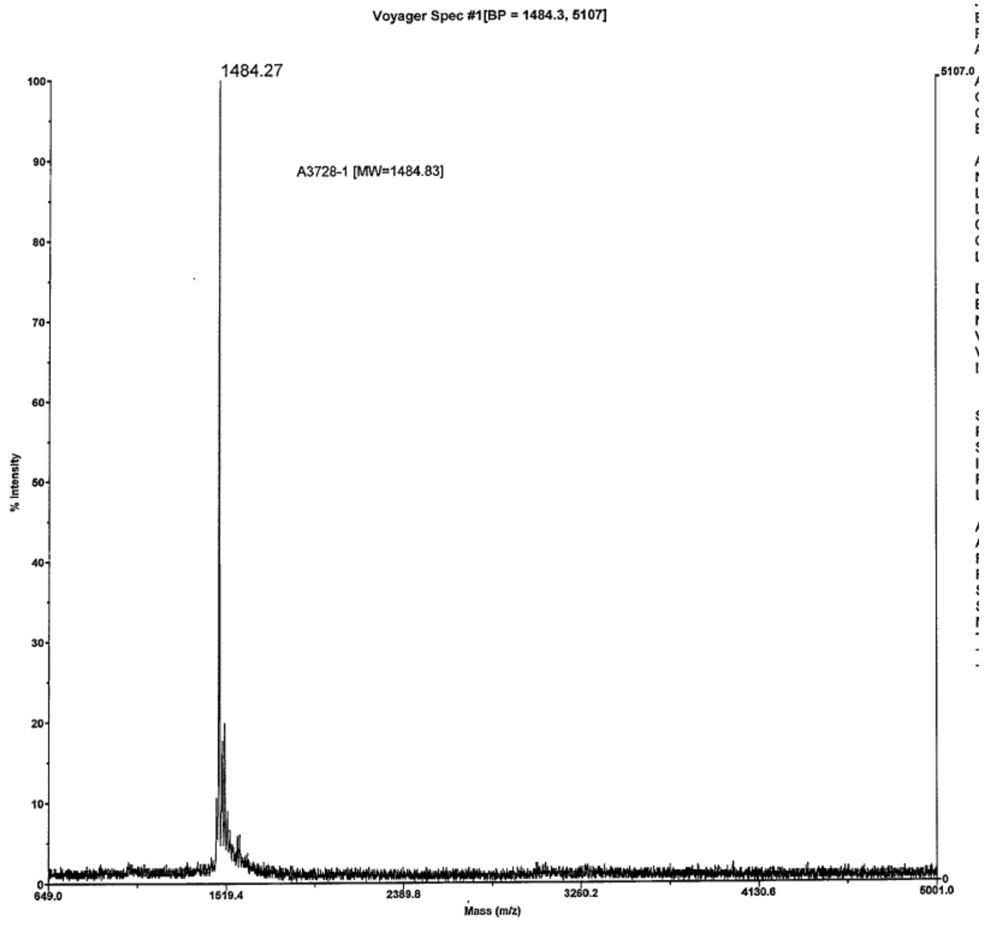
Voyager Spec #1[BP = 1484.3, 5107]
1484.27
A3728-1 [MW=1484.83]
5107.0
% Intensity
100
90
80
70
60
50
40
30
20
10
0
649.0
1519.4
2389.8
3260.2
4130.6
5001.0
Mass (m/z)

14aa-II

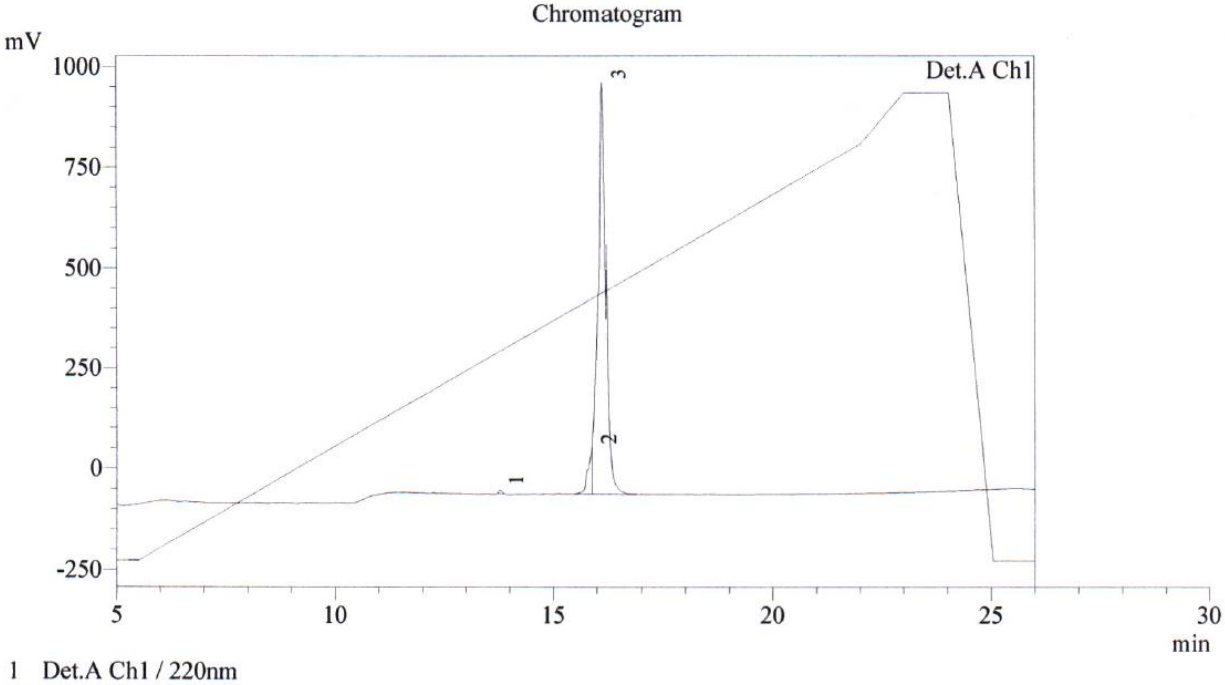

Chromatogram
mV
1000
750
500
250
0
-250
Det.A Ch1
3
2
1
5
10
15
20
25
30
min
1 Det.A Ch1 / 220nm

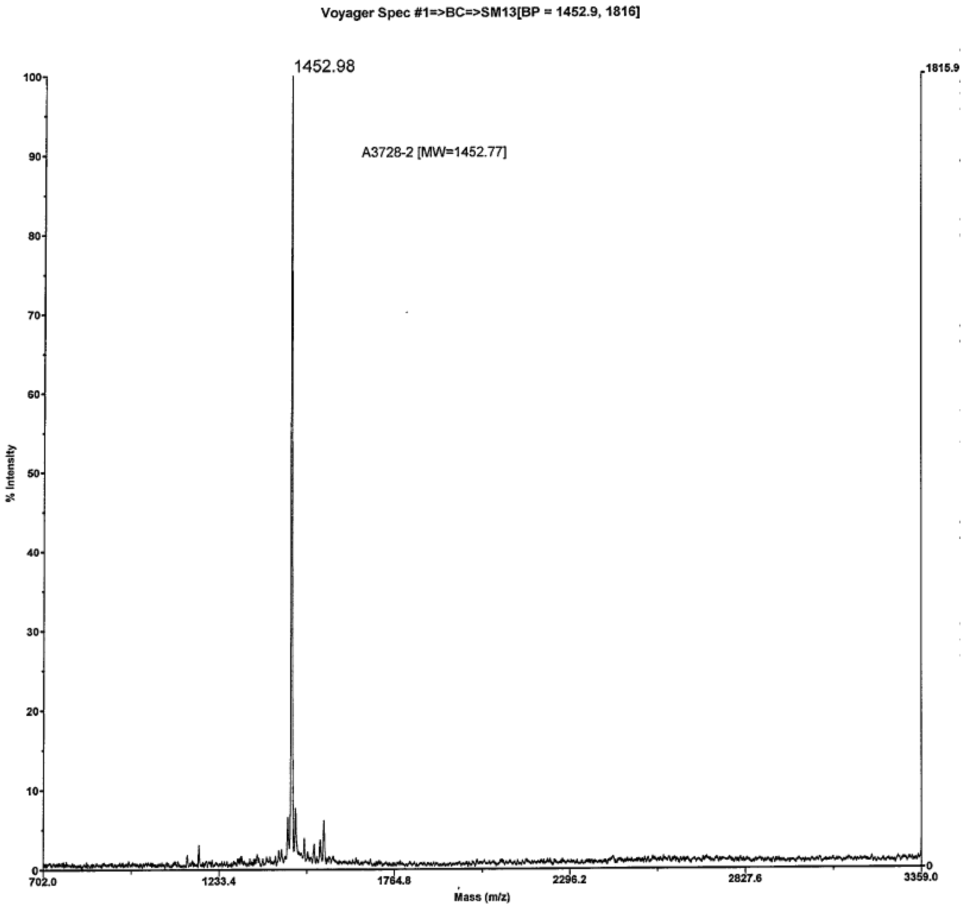

Voyager Spec #1=>BC=>SM13[BP = 1452.9, 1816]
1452.98
A3728-2 [MW=1452.77]
1815.9
% Intensity
Mass (m/z)
702.0
1233.4
1764.8
2296.2
2827.6
3359.0

13aa

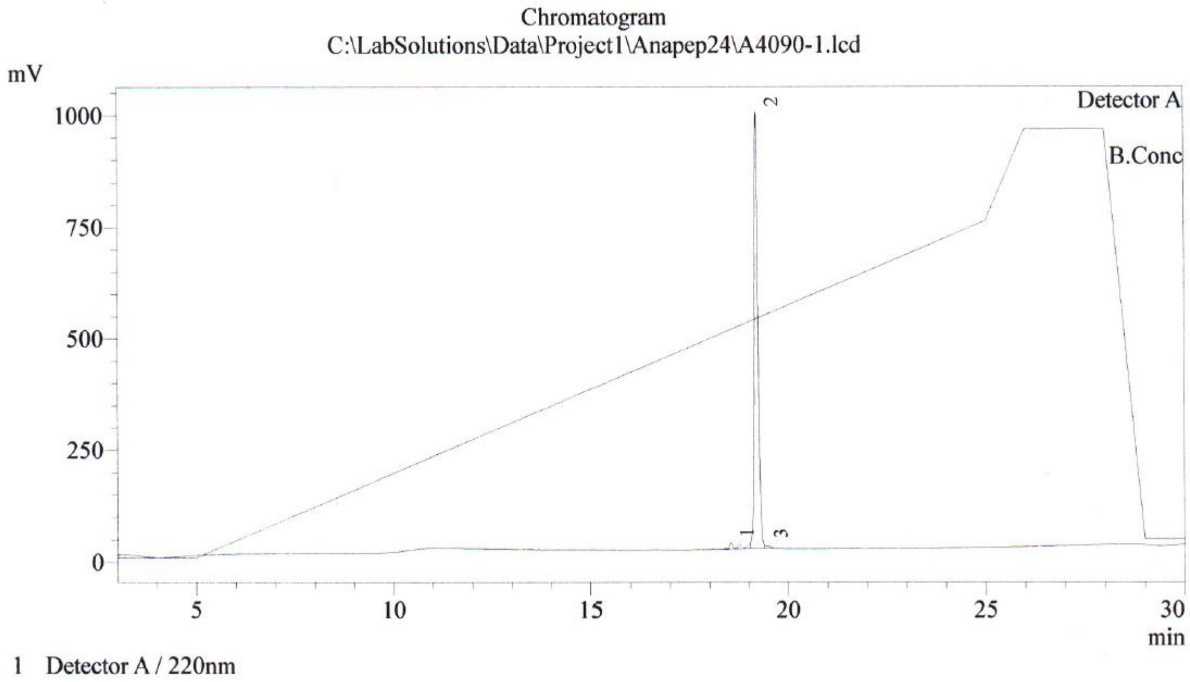

Chromatogram
C:\LabSolutions\Data\Project1\Anapep24\A4090-1.lcd
mV
1000
750
500
250
0
Detector A
B.Conc
1
2
3
5
10
15
20
25
30
min
1 Detector A / 220nm

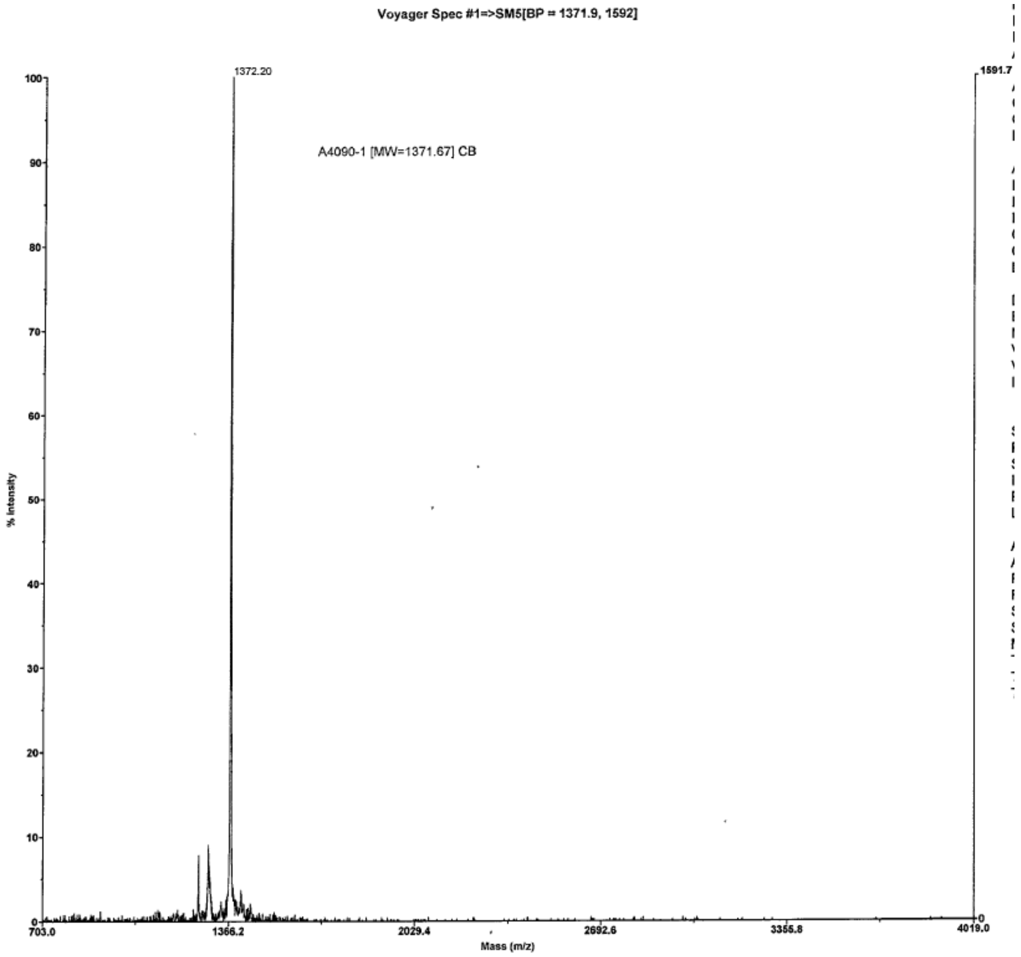

Voyager Spec #1=>SM5[BP = 1371.9, 1592]
1372.20
1591.7
A4090-1 [MW=1371.67] CB
% Intensity
100
90
80
70
60
50
40
30
20
10
0
703.0
1366.2
2029.4
2692.6
3355.8
4019.0
Mass (m/z)

12aa

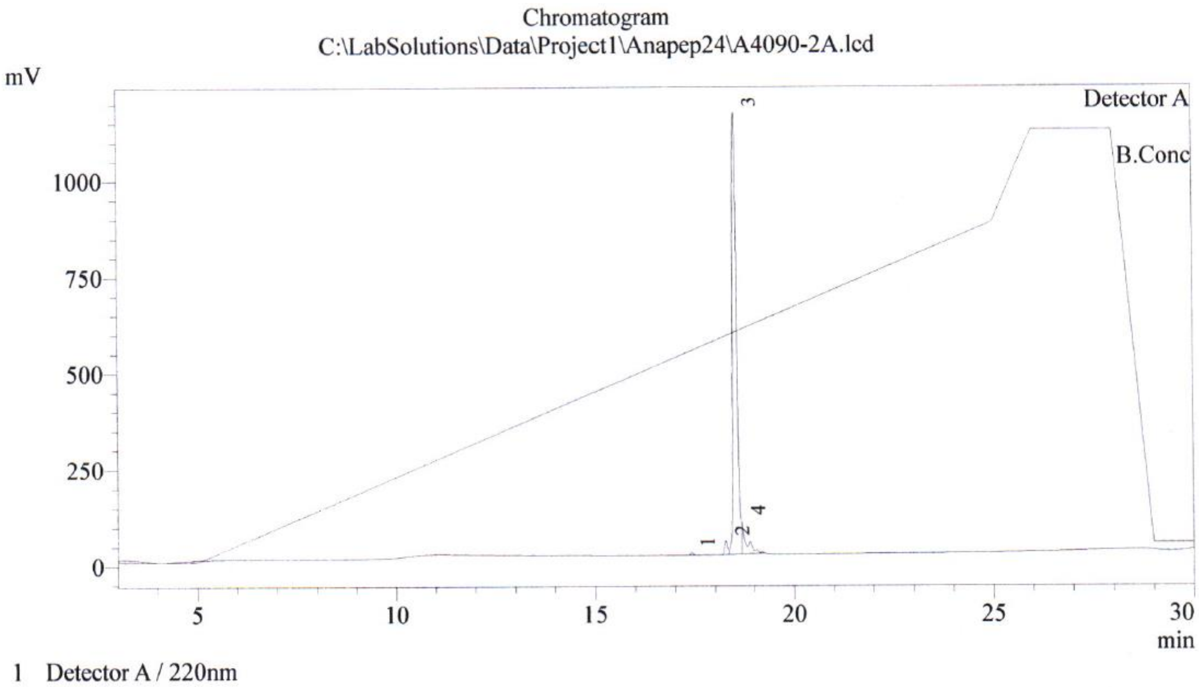
Chromatogram
C:\LabSolutions\Data\Project1\Anapep24\A4090-2A.lcd
mV
Detector A
B.Conc
1000
750
500
250
0
1
2
3
4
5
10
15
20
25
30
min
1 Detector A / 220nm

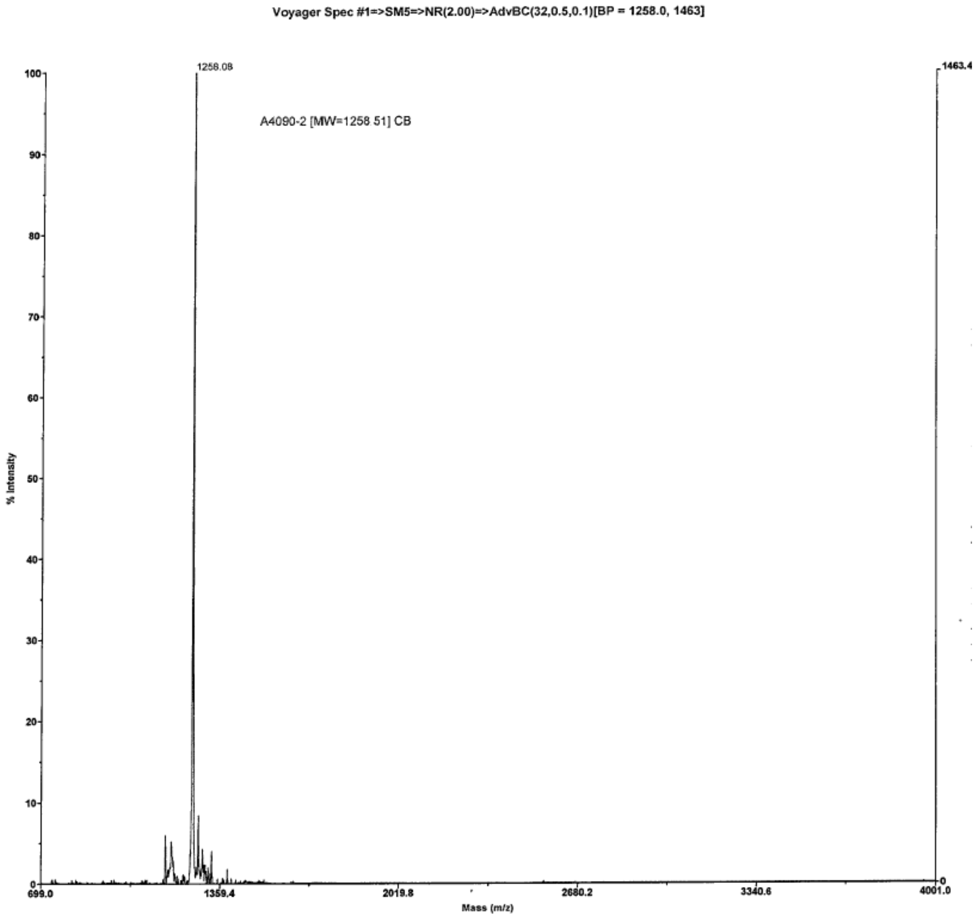
Voyager Spec #1=>SM5=>NR(2.00)=>AdvBC(32,0.5,0.1)[BP = 1258.0, 1463]
1258.08
1463.4
A4090-2 [MW=1258.51] CB
% Intensity
100
90
80
70
60
50
40
30
20
10
0
699.0
1359.4
2019.8
2680.2
3340.6
4001.0
Mass (m/z)

11aa

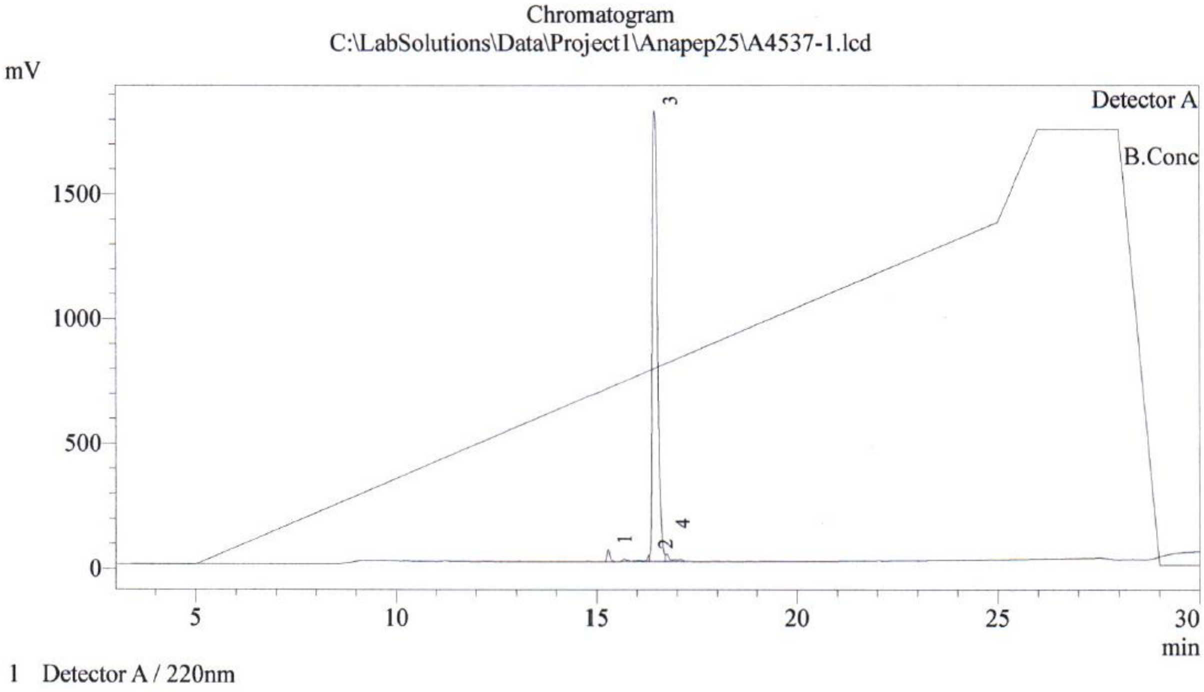

Chromatogram
C:\LabSolutions\Data\Project1\Anapep25\A4537-1.lcd
mV
Detector A
B.Conc
1500
1000
500
0
1
2
3
4
5
10
15
20
25
30
min
1 Detector A / 220nm

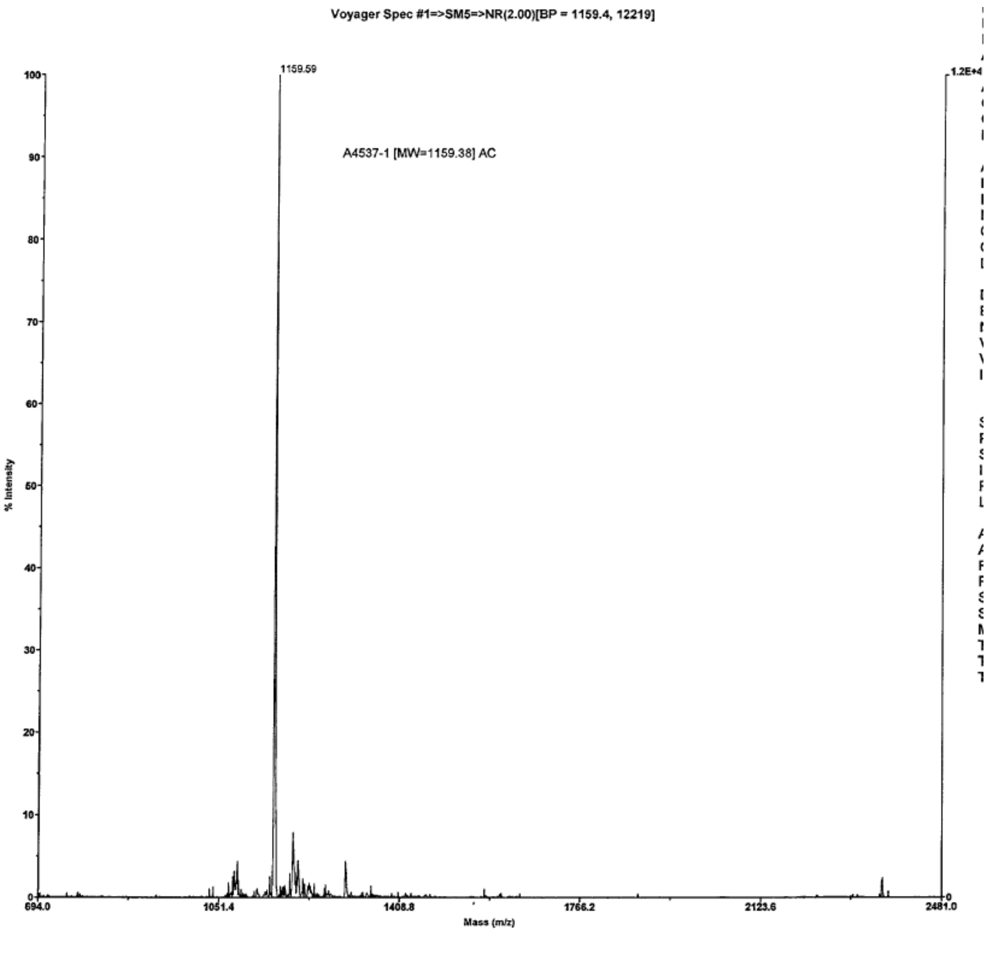

Voyager Spec #1=>SM5=>NR(2.00)[BP = 1159.4, 12219]
1159.59
A4537-1 [MW=1159.38] AC
1.2E+4
% Intensity
Mass (m/z)
694.0
1051.4
1408.8
1766.2
2123.6
2481.0

10aa

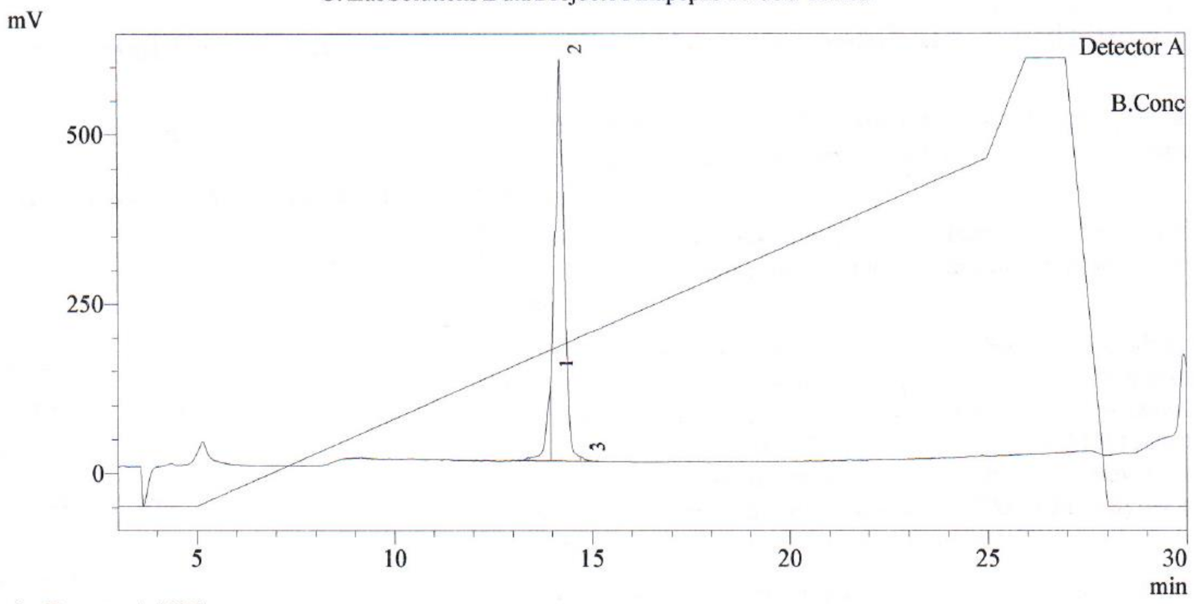

1 Detector A / 220nm

Data: A4950-1 [MW=1088.30] AC_0001:B4 27 August 2020 11:05:55 Cal:Custom Calibration by MALDISolutionsAdmin on 27 August 2020 11:08:02
Shimadzu MALDI-8020: Tuning Linear, Power 25, P.Ext at 2605.00 (bin 120)
Processed data (averaged) : 2.1 mV [sum=104.2 mV], Smoothed = 15, profiles # 1 - 50

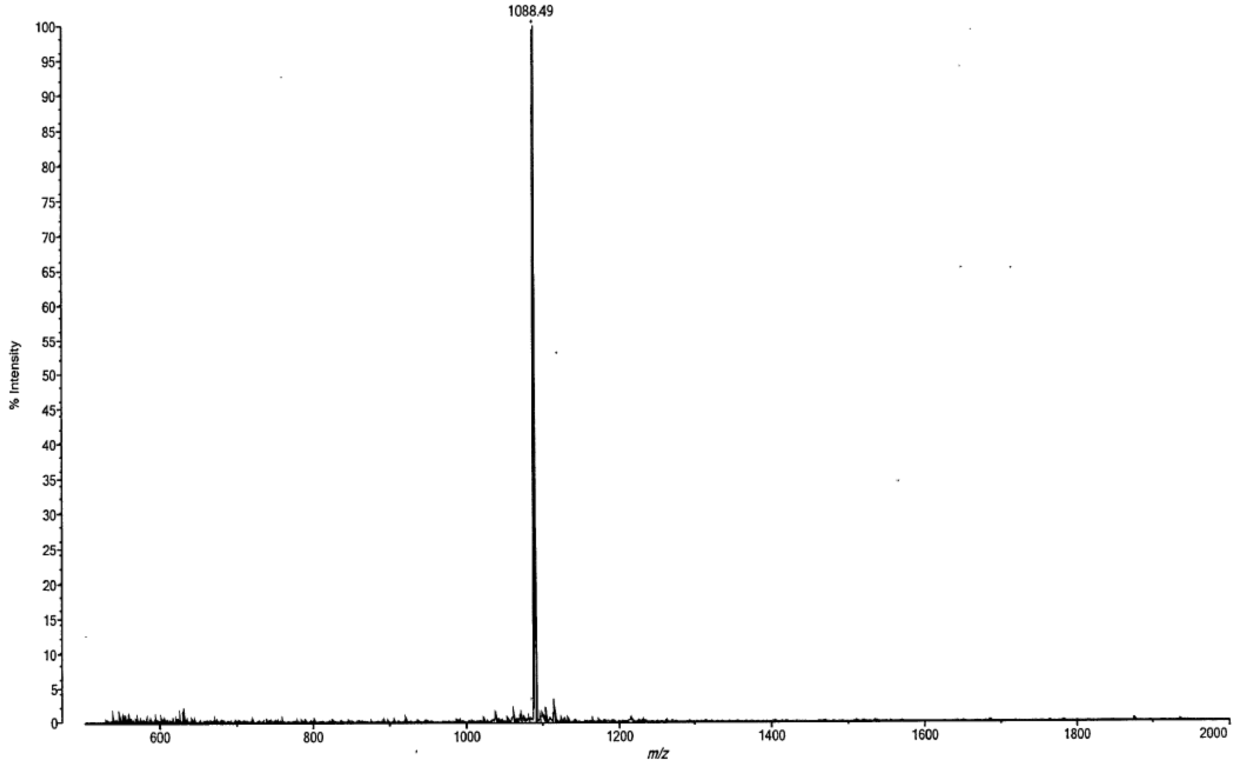

9aa

Chromatogram
C:\LabSolutions\Data\Project1\Anapep25\A4950-2A.lcd

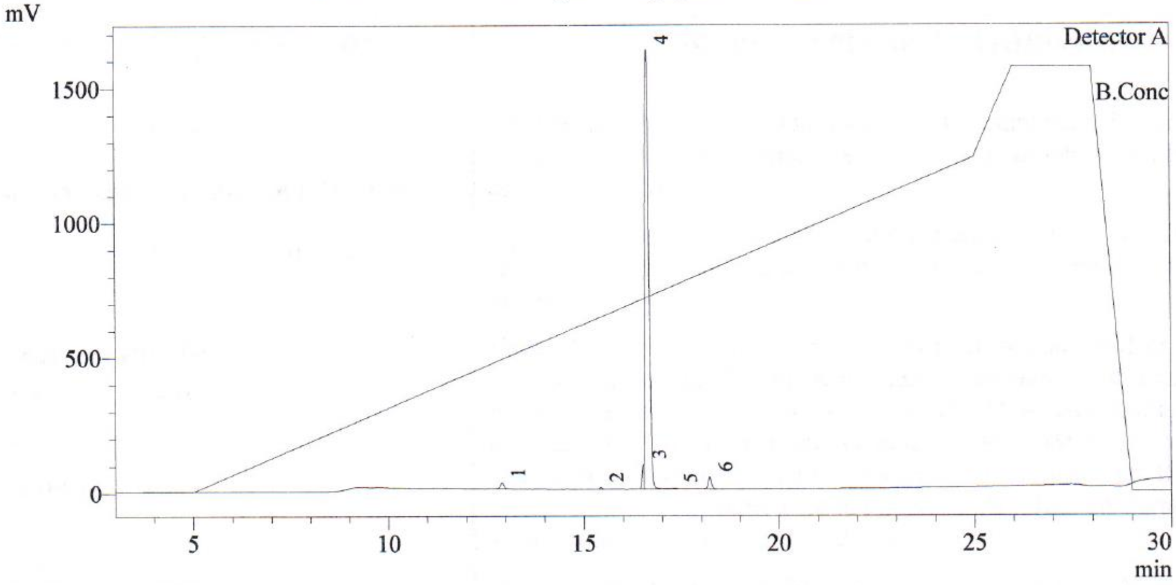

1 Detector A / 220nm

Data: A4950-2 [MW=1031.25] AC_0001:A4 27 August 2020 11:05:55 Cal:Custom Calibration by MALDISolutionsAdmin on 27 August 2020 11:09:47
Shimadzu MALDI-8020: Tuning Linear, Power 25, P.Ext at 2605.00 (bin 120)
Processed data (averaged) : 2.9 mV [sum=142.9 mV], Smoothed = 15, profiles # 1 - 50

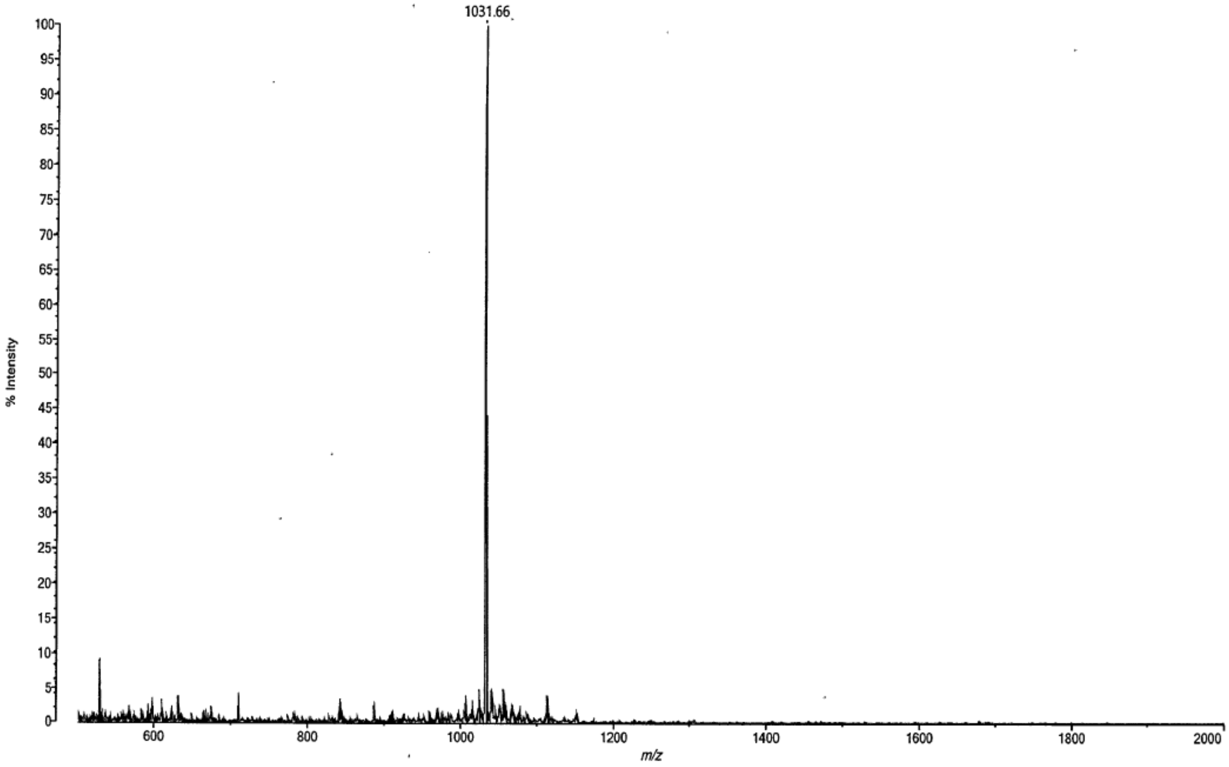

α7-EE

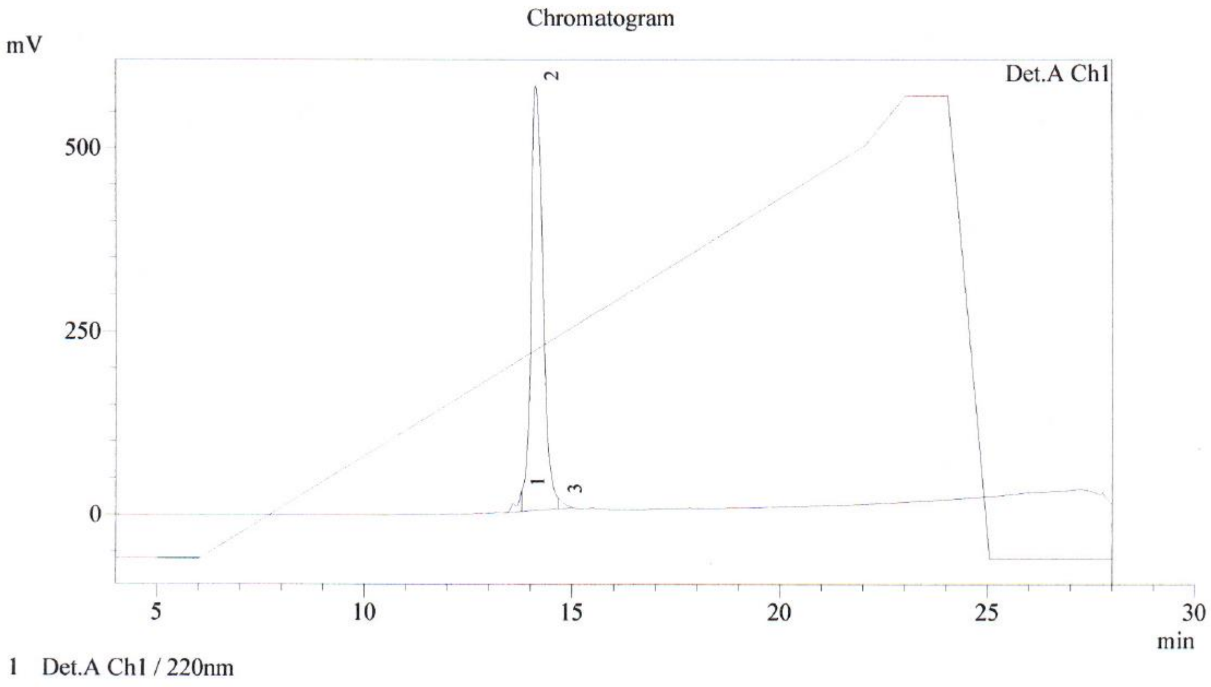

Chromatogram
mV
Det.A Ch1
500
250
0
2
1
3
5
10
15
20
25
30
min
1 Det.A Ch1 / 220nm

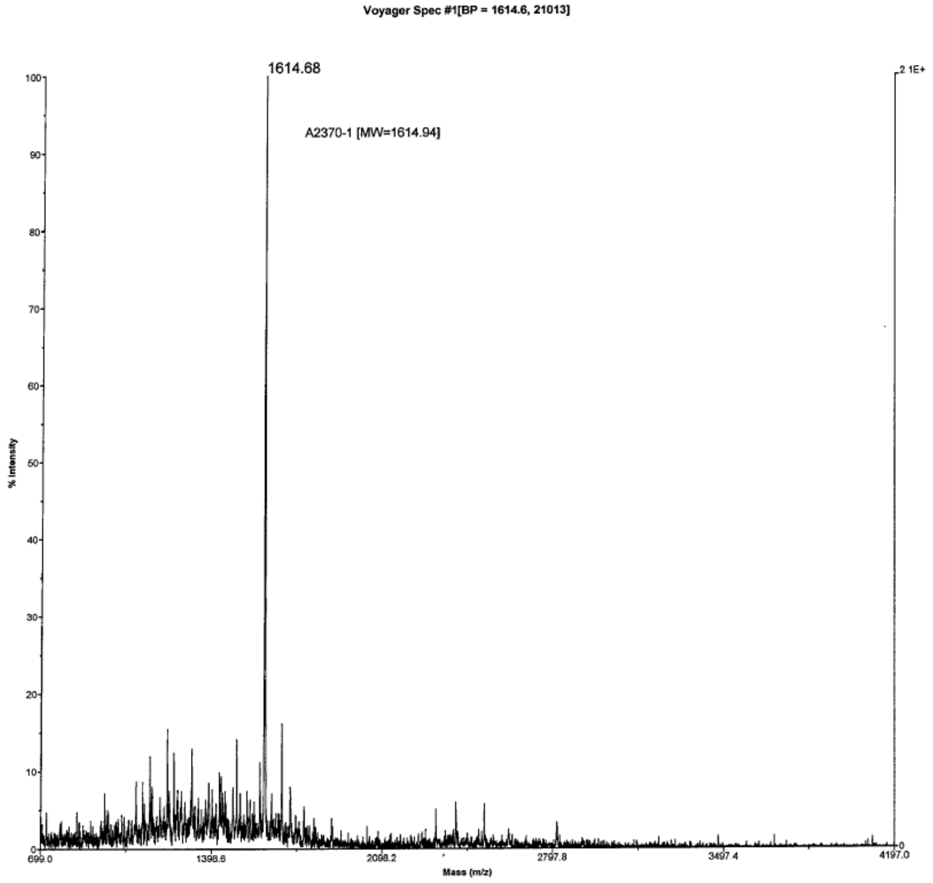

Voyager Spec #1[BP = 1614.6, 21013]
1614.68
A2370-1 [MW=1614.94]
% Intensity
Mass (m/z)
699.0
1398.6
2098.2
2797.8
3497.4
4197.0

α7-II

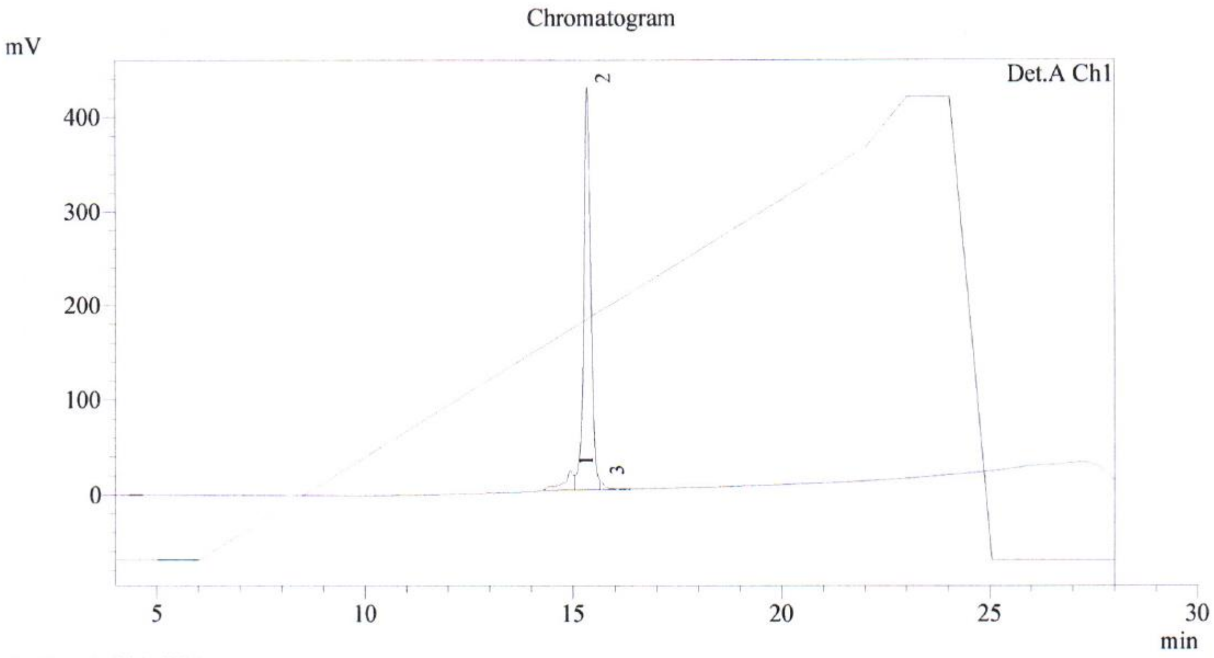

1 Det.A Ch1 / 220nm

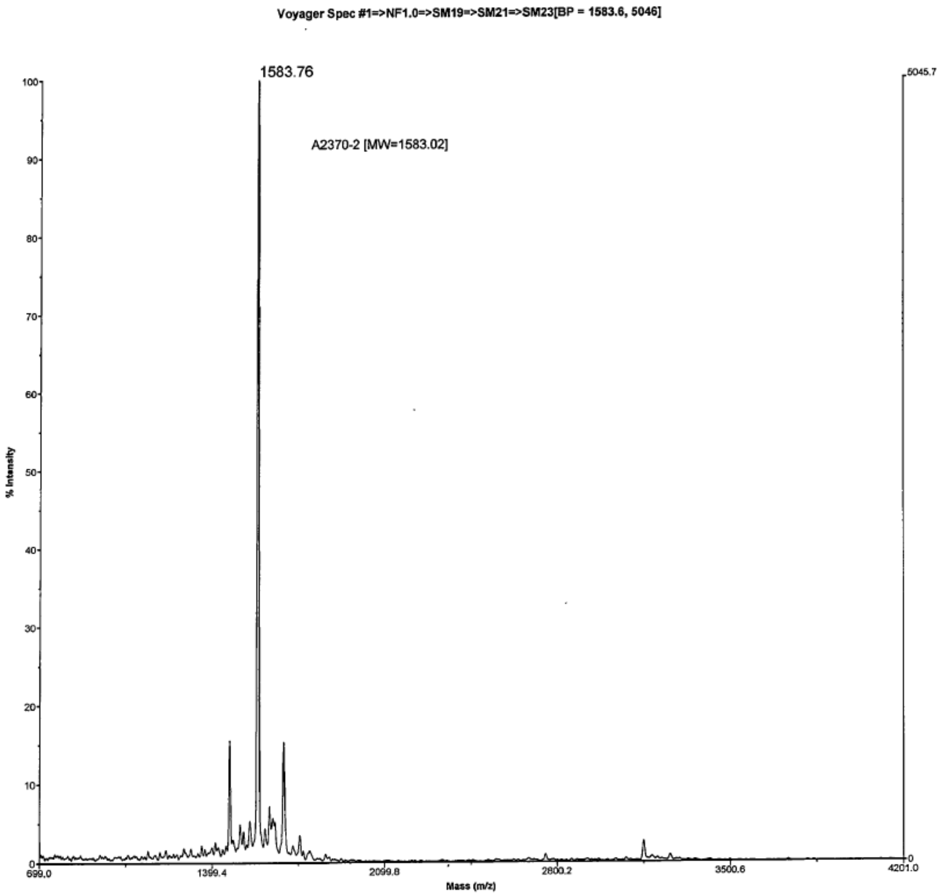

12aa-CtoS

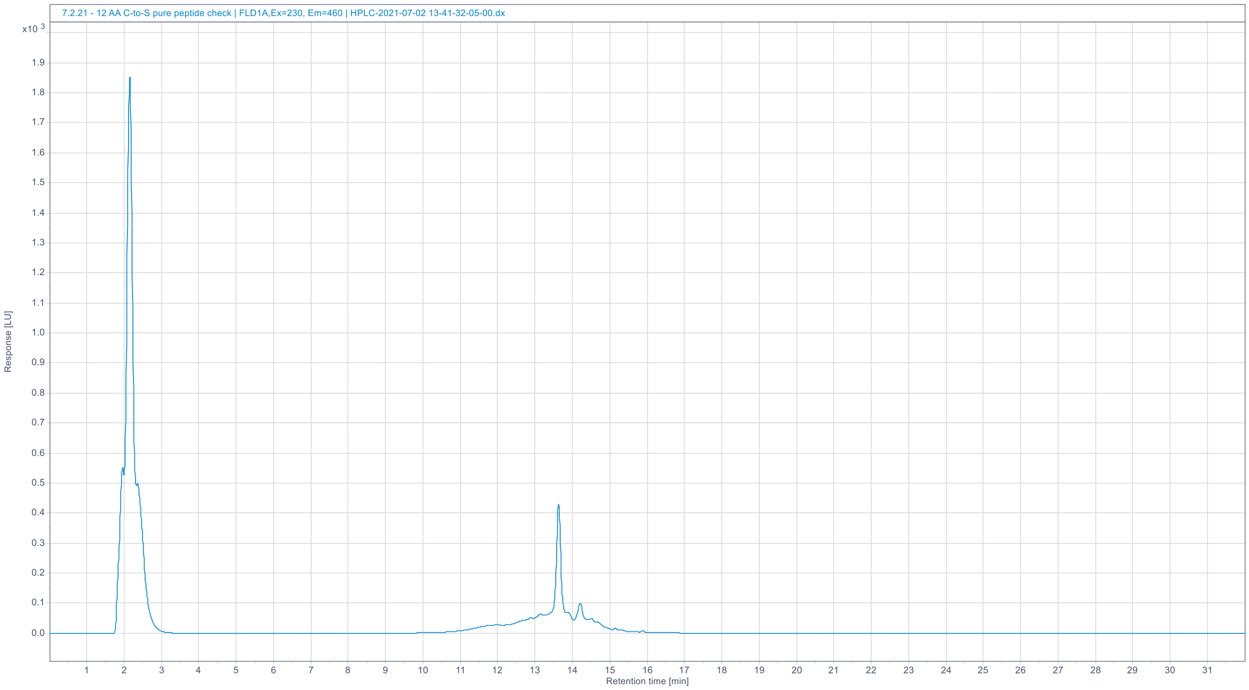

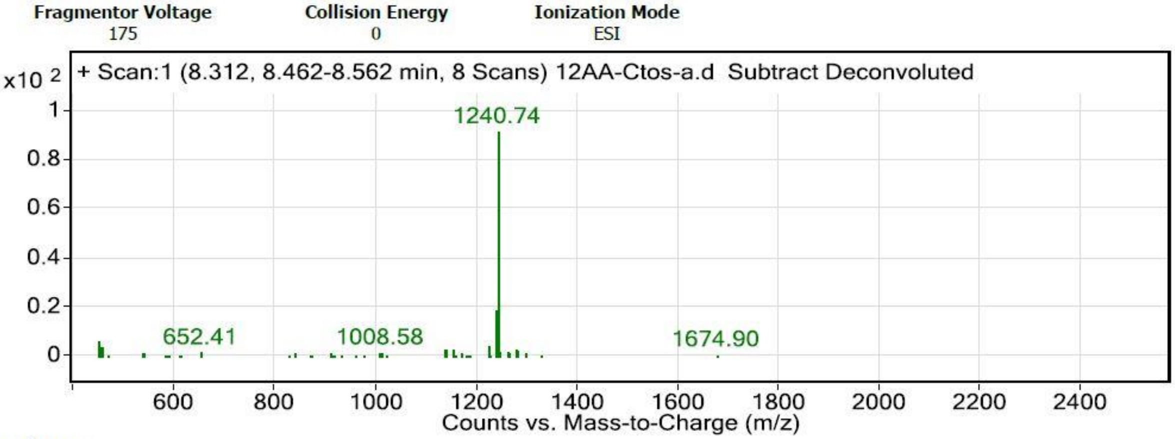

Peak List

m/z	Abund
451.22	9551.9
456.17	6375.1
1223.71	6649.6
1240.69	29821.5
1240.74	145934.6
1241.69	20043.2
1241.74	102582.2
1242.7	7643.5
1242.74	39640.6
1243.75	11235.6

12aa-ALK

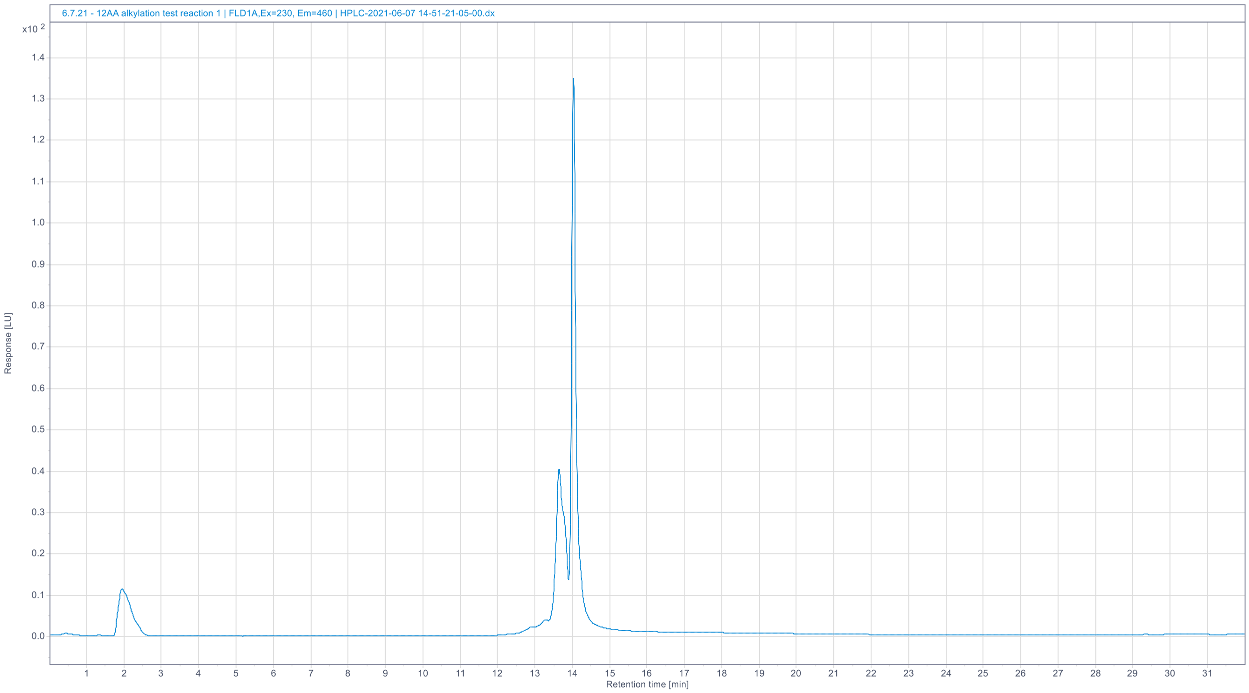

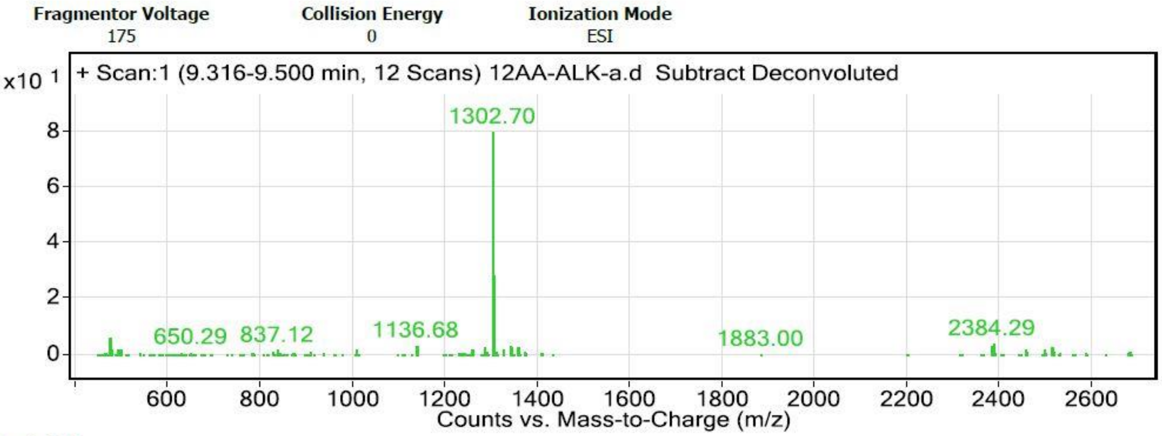

Peak List

m/z	Abund
476.24	3393.6
1302.65	11376.4
1302.7	43929.8
1303.66	8804.3
1303.7	31902.7
1304.66	4084.4
1304.7	15701.7
1305.7	6313.9
