# Supplementary material for: Structural and functional analysis of EntV reveals a 12 amino acid fragment protective against fungal infections
Source: Nat Commun. 2022 Oct 13;13:6047. doi: 10.1038/s41467-022-33613-1 (PMC9562342; doi:10.1038/s41467-022-33613-1)
Supplement: Supplementary file 5 — Reporting Summary [file 41467_2022_33613_MOESM5_ESM.pdf]

nature portfolio

Corresponding author(s): Danielle A. Garsin, Michael C. Lorenz

Last updated by author(s): Sep 9, 2022



# Reporting Summary

Nature Portfolio wishes to improve the reproducibility of the work that we publish. This form provides structure for consistency and transparency in reporting. For further information on Nature Portfolio policies, see our Editorial Policies and the Editorial Policy Checklist.

## Statistics

For all statistical analyses, confirm that the following items are present in the figure legend, table legend, main text, or Methods section.

| n/a | Confirmed | |
|---|---|---|
| ☐ | ☒ | The exact sample size (*n*) for each experimental group/condition, given as a discrete number and unit of measurement |
| ☐ | ☒ | A statement on whether measurements were taken from distinct samples or whether the same sample was measured repeatedly |
| ☐ | ☒ | The statistical test(s) used AND whether they are one- or two-sided<br>*Only common tests should be described solely by name; describe more complex techniques in the Methods section.* |
| ☐ | ☒ | A description of all covariates tested |
| ☐ | ☒ | A description of any assumptions or corrections, such as tests of normality and adjustment for multiple comparisons |
| ☐ | ☒ | A full description of the statistical parameters including central tendency (e.g. means) or other basic estimates (e.g. regression coefficient) AND variation (e.g. standard deviation) or associated estimates of uncertainty (e.g. confidence intervals) |
| ☐ | ☒ | For null hypothesis testing, the test statistic (e.g. *F*, *t*, *r*) with confidence intervals, effect sizes, degrees of freedom and *P* value noted<br>*Give P values as exact values whenever suitable.* |
| ☒ | ☐ | For Bayesian analysis, information on the choice of priors and Markov chain Monte Carlo settings |
| ☒ | ☐ | For hierarchical and complex designs, identification of the appropriate level for tests and full reporting of outcomes |
| ☒ | ☐ | Estimates of effect sizes (e.g. Cohen's *d*, Pearson's *r*), indicating how they were calculated |

*Our web collection on statistics for biologists contains articles on many of the points above.*

## Software and code

Policy information about availability of computer code

| | |
|---|---|
| Data collection | N/A |
| Data analysis | All statistical analysis in the manuscript was performed using Graphpad Prism 9.0. |

For manuscripts utilizing custom algorithms or software that are central to the research but not yet described in published literature, software must be made available to editors and reviewers. We strongly encourage code deposition in a community repository (e.g. GitHub). See the Nature Portfolio guidelines for submitting code & software for further information.

## Data

Policy information about availability of data

All manuscripts must include a data availability statement. This statement should provide the following information, where applicable:

- Accession codes, unique identifiers, or web links for publicly available datasets
- A description of any restrictions on data availability
- For clinical datasets or third party data, please ensure that the statement adheres to our policy

All data are available in the main text, supplementary materials, or the source data file except for the structural data that was deposited to the Protein Data Bank under the accession code 7ROA.

## Human research participants

Policy information about studies involving human research participants and Sex and Gender in Research.

| | |
|---|---|
| Reporting on sex and gender | N/A |
| Population characteristics | N/A |
| Recruitment | N/A |
| Ethics oversight | N/A |

Note that full information on the approval of the study protocol must also be provided in the manuscript.

# Field-specific reporting

Please select the one below that is the best fit for your research. If you are not sure, read the appropriate sections before making your selection.

[x] Life sciences   [ ] Behavioural & social sciences   [ ] Ecological, evolutionary & environmental sciences

For a reference copy of the document with all sections, see nature.com/documents/nr-reporting-summary-flat.pdf

# Life sciences study design

All studies must disclose on these points even when the disclosure is negative.

| | |
|---|---|
| Sample size | For vertebrate animal experiments group sizes were determined using power calculations with assumptions about variation and thresholds for significance based on our previous experience with these models. |
| Data exclusions | No exclusions. |
| Replication | All C. elegans experiments were replicated two or three times. The biofilm data was replicated three times. Due to cost and ethics the vertebrate animal experiments were not replicated exactly, but many of the same experimental groups were used in multiple experiments. For example the antifungal efficacy of the 12mer, the main point of the manuscript, was replicated in separate OPC model experiments shown in Figures 5a, 5c, 5d and 5e. No replicates were excluded. |
| Randomization | Allocation of samples/organisms was random. |
| Blinding | Investigators were not always blinded due to a lack of sufficient personnel. However, most of the manuscript's data collection involved objective rather and subjective measurements such as readings from instruments or live/dead scoring. For the vertebrate animal experiments, investigators were blinded with records held by the PIs. The exception was the OPC model where the personnel handling the animals were not blinded (so they could ensure proper dosing of the groups). However, the individual who processed the tissue samples was blinded to the identity of the groups. |

# Reporting for specific materials, systems and methods

We require information from authors about some types of materials, experimental systems and methods used in many studies. Here, indicate whether each material, system or method listed is relevant to your study. If you are not sure if a list item applies to your research, read the appropriate section before selecting a response.

## Materials & experimental systems

| n/a | Involved in the study | |
|---|---|---|
| [x] | [ ] | Antibodies |
| [x] | [ ] | Eukaryotic cell lines |
| [x] | [ ] | Palaeontology and archaeology |
| [ ] | [x] | Animals and other organisms |
| [x] | [ ] | Clinical data |
| [x] | [ ] | Dual use research of concern |

## Methods

| n/a | Involved in the study | |
|---|---|---|
| [x] | [ ] | ChIP-seq |
| [x] | [ ] | Flow cytometry |
| [x] | [ ] | MRI-based neuroimaging |

## Animals and other research organisms

Policy information about studies involving animals; ARRIVE guidelines recommended for reporting animal research, and Sex and Gender in Research

| | |
|---|---|
| Laboratory animals | Mice and rats were used in these studies. The OPC experiments used BALB/c mice,10 wks, 18-20 g and the disseminated infections used female ICR mice, 6 wks, 18-20 g. The catheter model used 400g, 16 wk old, female Sprague-Dawley rats.<br>Animals were kept in facilities with a 12 hour light-dark cycle at 22°C, and with ambient humidity.<br>As 1000-celled, hermaphroditic invertebrates, C. elegans are not covered by animal study guidelines. But to be complete, we used Caenorhabditis elegans strain glp-4(bn2); sek-1(km-4) hermaphrodites and infections were initiated at Larval Stage 4. Animals were incubated in incubators without light at 20°C and ambient humidity during development, and at 25°C once infected. |
| Wild animals | No wild animals were used in this study. |
| Reporting on sex | Sex was not considered in the study design except for the OPC model in which an initial experiment was carried out with both sexes and then disaggregated by sex (see Figure 5A and B and extended Figure 5F and G). As there were no sex-based differences, female animals were used in the follow-up experiments due to increased ease of handling. Female rats were used. |
| Field-collected samples | No field collected samples were used in this study. |
| Ethics oversight | The vertebrate animal experiments were conducted under protocols approved by the Animal Welfare Committees of the University of Wisconsin (rat catheter) or University of Texas Health Science Center at Houston (all others) |

Note that full information on the approval of the study protocol must also be provided in the manuscript.
